# Stimuli-responsive nanoformulations for CRISPR-Cas9 genome editing

**DOI:** 10.1186/s12951-022-01570-y

**Published:** 2022-08-02

**Authors:** Tianxu Fang, Xiaona Cao, Mysha Ibnat, Guojun Chen

**Affiliations:** 1 Department of Biomedical Engineering, McGill University, Montreal, QC H3G 0B1 Canada; 2 Rosalind & Morris Goodman Cancer Institute, McGill University, Montreal, QC H3G 0B1 Canada; 3 School of Nursing, Tianjin Medical University, Tianjin, China

**Keywords:** Nanoformulations, CRISPR-Cas9, Stimuli-responsive, Genome editing, External stimuli, Internal stimuli, Drug delivery

## Abstract

The CRISPR-Cas9 technology has changed the landscape of genome editing and has demonstrated extraordinary potential for treating otherwise incurable diseases. Engineering strategies to enable efficient intracellular delivery of CRISPR-Cas9 components has been a central theme for broadening the impact of the CRISPR-Cas9 technology. Various non-viral delivery systems for CRISPR-Cas9 have been investigated given their favorable safety profiles over viral systems. Many recent efforts have been focused on the development of stimuli-responsive non-viral CRISPR-Cas9 delivery systems, with the goal of achieving efficient and precise genome editing. Stimuli-responsive nanoplatforms are capable of sensing and responding to particular triggers, such as innate biological cues and external stimuli, for controlled CRISPR-Cas9 genome editing. In this Review, we overview the recent advances in stimuli-responsive nanoformulations for CRISPR-Cas9 delivery, highlight the rationale of stimuli and formulation designs, and summarize their biomedical applications.

## Introduction

The past few years have seen exponential growth in genome editing research worldwide due to the development of CRISPR-Cas9 technology. The 2020 Nobel Prize in Chemistry was awarded for CRISPR-Cas9, highlighting its extraordinary potential in genome editing [1, 2]. This emerging platform has shown tremendous promise in many applications, ranging from basic biology research to bioengineering, food sciences, and healthcare [3–9]. Given the international recognition of this technology, global markets for CRISPR-Cas9 genome editing-related products have also been explosively expanded [10].

CRISPR-Cas9 genome editing technology relies on the Cas9 nuclease to cut double-strand DNA and the pre-designed single-guide RNA (sgRNA) to navigate Cas9 to the gene locus of interest [11–14]. The process of breaking the double-strand in DNA is followed by either the nonhomologous end-joining (NHEJ) or homology-directed repair (HDR), for specific gene knock-out or knock-in, respectively [14–20]. Compared to other conventional genome editing platforms, such as zinc finger nucleases (ZFNs) and transcription activator-like nucleases (TALEN), the CRISPR-Cas9 system possesses several advantages [21]. First, in the CRISPR-Cas9 system, sgRNAs (about 100 base pairs in length) can be easily custom-designed for targeted genes, which is much simpler compared with ZFN- or TALEN-based tools that require sophisticated design and synthesis of a bulky guiding protein (zinc finger DNA-binding domain and transcription activator-like effector) [22–25]. Second, CRISPR-Cas9 technology offers higher precision in genome editing compared to the other two methods [26–28]. Third, the CRISPR-Cas9 system offers possibilities for editing at multiple genetic loci at the same time (multiplexing), which cannot be achieved by other existing technologies [26, 29].

The CRISPR-Cas9 technology can be realized in the form of either plasmid DNA that encodes both Cas9 protein and sgRNA, CRISPR mRNA/sgRNA, or ribonucleoprotein (RNP, the complex of Cas9 protein and sgRNA) [30–34]. These three formats have their unique characteristics [35]. The DNA format has relatively high stability and can offer long-term editing effects, while it is accompanied by possible insertional mutagenesis, slow responses, high off-target effects, and low efficiency [36–38]. Utilization of mRNA avoids insertional mutagenesis, whereas inherent instability of mRNA may reduce overall editing efficiency [36, 39, 40]. RNP shows improved stability, rapid action, low off-target effects, low immunogenicity, and no risk of insertional mutagenesis, despite its high cost and relatively short gene editing duration [36, 41, 42]. Therefore, depending on specific applications and requirements, all these three formats have been widely explored for many biomedical applications, including therapies for cancer as well as genetic, infectious, and immunological diseases [14, 43–46].

It is well-known that DNA, mRNA, and RNP are all highly negatively-charged molecules, which results in extremely low intracellular delivery due to electrostatic repulsion by the negatively-charged cell membranes [35, 47–50]. Therefore, engineering strategies to enable efficient intracellular delivery of CRISPR-Cas9 components have been a central theme for broadening the impact of the CRISPR-Cas9 technology. Currently, both viral and non-viral vectors have been deployed for CRISPR-Cas9 delivery [51]. Viral vectors, such as adeno-associated viruses (AAVs) and lentiviral vectors (LVs), are commonly used carriers to transport genome editing systems intracellularly with high efficiency, while their applications are restricted owing to the risks of immunogenicity and mutagenesis, as well as unsatisfying loading capacities [52–54]. In addition, viral vectors offer long-term genome editing activities in cells, which could result in increased off-target effects [55–57].

**Fig. 1:**
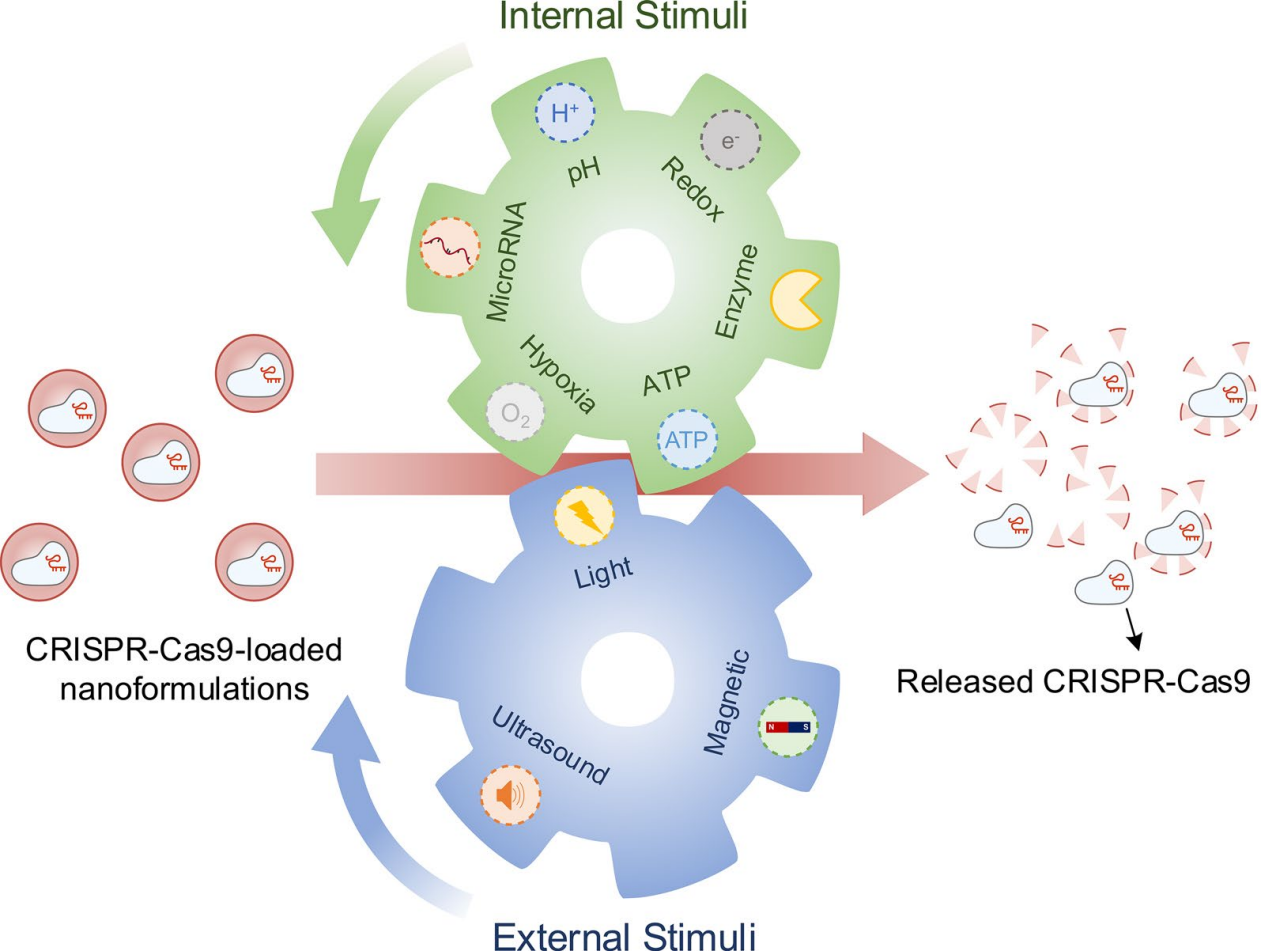
Scheme of internal and external stimuli-responsive nanoformulations for CRISPR-Cas9 genome editing

Non-viral Cas9 delivery *via* nanoparticles (NPs) hold promise to overcome these limitations [39, 58–61]. Several unique NPs have been reported for CRISPR-Cas9 delivery, including lipid, polymeric, silicon, and gold nanoparticles. These non-viral NPs generally show improved biocompatibility and can enhance CRISPR-Cas9 delivery efficiency into cells both in vitro and in vivo [62, 63]. Nevertheless, there are still two critical issues associated with non-viral CRISPR-Cas9 delivery. First, the efficiency of non-viral delivery systems remains much lower compared to the viral vectors [41, 64]. The main reason is that, similar to other nanomedicines, delivery of CRISPR-Cas9 using NPs needs to overcome several extracellular and intracellular barriers [62, 65, 66]. Specifically, extracellular barriers include unwanted interactions with serum proteins, rapid clearance by immune cells, limited diffusion through extracellular spaces and tight junctions, and low cell targeting capabilities; intracellular barriers mainly involve rapid degradation in endo/lysosomes, lack of active payload release mechanisms, and poor nucleus translocation [67, 68]. Second, active delivery strategies to achieve precise genome editing are still less developed [69]. Therefore, in order to address these challenges and achieve efficient and precise genome editing, researchers have been investigating stimuli-responsive nanoplatforms, the next-generation non-viral delivery system, for CRISPR-Cas9 delivery. Stimuli-responsive nanoplatforms are capable of sensing and responding to particular triggers for spatiotemporal control of drug delivery. These triggers include innate biological cues (e.g., pH, enzymes, ATP, RNA, redox, and oxygen levels) and external stimuli (e.g., light, ultrasound and magnetism) [70–74]. In this review, we review the development of stimuli-responsive nanoformulations for spatiotemporally controllable delivery of the CRISPR-Cas9 system, highlight the rationale of formulation designs, and summarize their biomedical applications (Fig. 1).

### Internal stimuli-responsive nanoformulations for CRISPR-Cas9 delivery

With recent advances in biosciences, materials sciences, and bioengineering, intelligent bioresponsive drug delivery systems leveraging internal stimuli have already made an enormous impact on medical technology, greatly improving the performance of many existing drugs and enabling the use of entirely new therapies [75–81]. It offers exciting opportunities for CRISPR-Cas-based genome editing technology in the context of efficiency and precision. The concept of smart responsive drug delivery was first introduced in the late 1970s with the use of thermo-responsive liposomes for the controlled drug release through hyperthermia [82]. Since then, ample research has been focused on smart materials, which are capable of sensing and responding to particular biological cues for spatiotemporal drug delivery, exponentially emerging as a panacea for clinical use in patients [83–86]. These internal biological stimuli mostly include innate cues (e.g., pH, enzymes, ATP, glucose, redox, and oxygen levels) and pathological abnormalities in distinct types of diseases (such as cancer, autoimmune disorders, degenerative diseases, infections, and cardiovascular diseases) [70–74]. In this section, we will highlight the progress in developing internal stimuli-responsive nanoformulations for bioresponsive CRISPR-Cas9 genome editing, where CRISPR-Cas9 can be delivered in the form of DNA, mRNA, and protein complex. It should be noted that these formulations can be also applied to other biotherapeutics, including but not limited to DNA, RNA and proteins [87–89].

### pH-responsive CRISPR-Cas9 delivery

pH values vary in specific organs (stomach (pH 1.5–3.5), small intestine (pH 5.5–6.8), the colon (6.4–7.0), and the vagina (pH 3.8–4.5)), intracellular compartments (endosomes or lysosomes (pH 4.0–6.5)), and disease environment (cancer or inflammatory sites) [90, 91]. These environmental pH differences have been extensively exploited for the design of bio-responsive drug delivery systems [92, 93]. One critical intracellular barrier for CRISPR-Cas9 delivery lies in endo/lysosomes where many enzymes and low pHs will quickly degrade CRISPR-Cas9 components [94]. Given the acidity nature of endo/lysosomes, many pH-responsive nanoformulations have been engineered for CRISPR-Cas9 delivery, aiming to facilitate endo/lysosomal escape of nanoformulations or payloads.

**Fig. 2:**
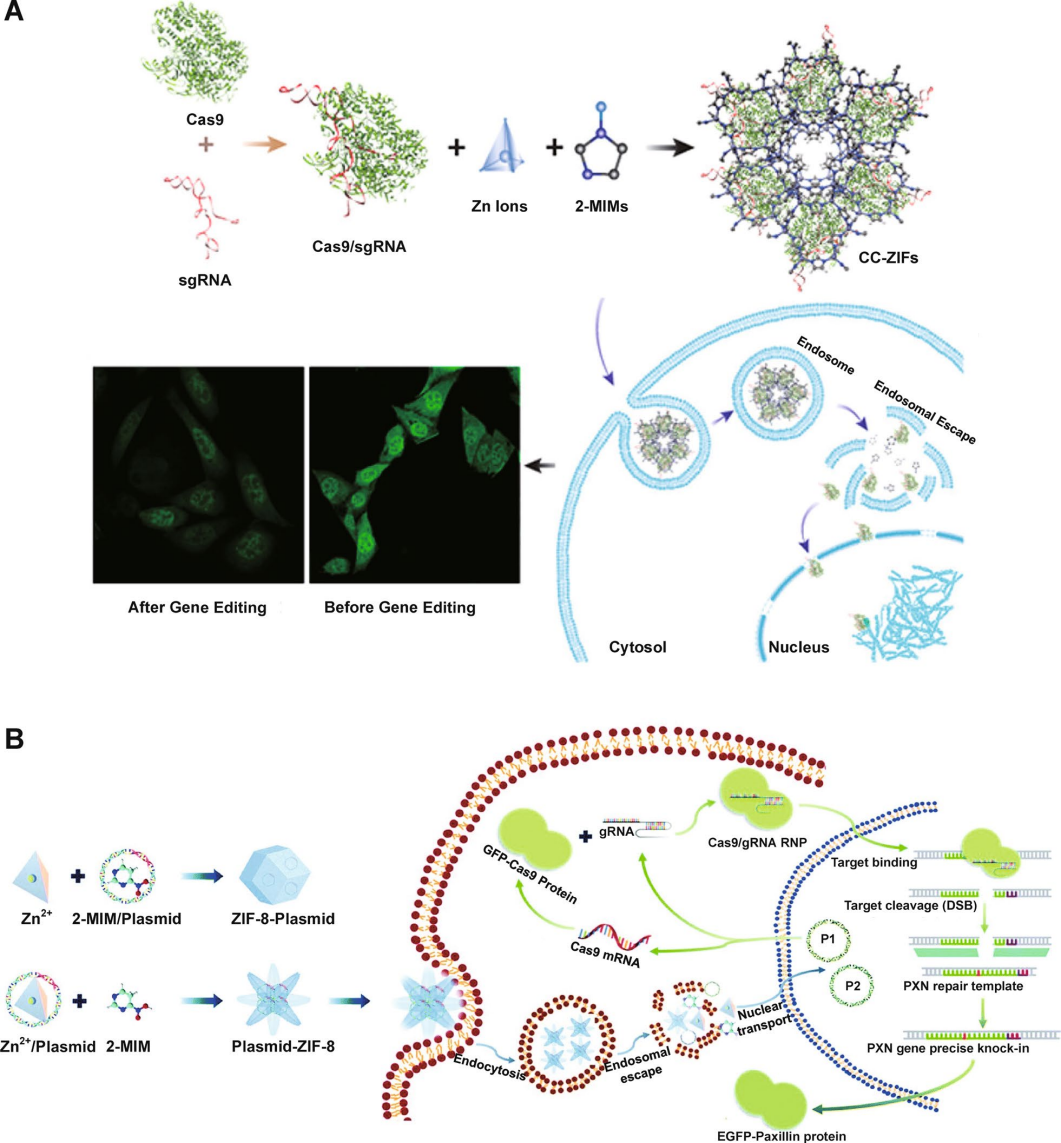
Imidazole-based pH-responsive CRISPR-Cas9 delivery nanoformulations. **A** Encapsulation of the negatively charged Cas9/sgRNA within positively charged ZIF-8 to form CC-ZIFs, endosomal escape of CC-ZIFs, and laser scanning confocal microscope images of cells before and after treatment with CC-ZIFs. Reprinted (adapted) with permission from [98]. Copyright 2022 American Chemical Society. **B** ZIF-8-based nanocarrier for the delivery of CRISPR-Cas9 plasmids: preparation of CRISPR-Cas9 plasmid-loaded nanostructures, intracellular delivery of the two kinds of plasmids to the nucleus for paxillin gene knock-in. Reproduced from Ref. [99] with permission from the Royal Society of Chemistry

Imidazole with an alkalescent N atom is one of the most commonly used pH-responsive materials in the molecular structure and triggers the proton sponge effect for rapid endosomal escape [95]. Specifically, the protons in endosomes (pH < 6.5) are absorbed by alkalescent N atoms (pKa: ~6.5–6.9), leading to chlorine ions and water flowing into endosomes in order to balance endo/lysosomal membrane charges, eventually causing an osmotic pressure and burst of endosomes [96, 97]. Alsaiari et al. [98] reported nanoscaled zeolitic imidazole-containing frameworks (ZIFs) to encapsulate CRISPR-Cas9 (Fig. 2A). Cas9 and sgRNA were mixed with 2-methyl**imidazole** (2-MIM) and zinc nitrate in solution to prepare ZIF-8 co-encapsulating Cas9 protein and sgRNA (CC-ZIFs) in which Zn^2+^ and 2-MIM) formed the metal-organic framework (MOF) *via* coordination bonds. The imidazolyl protonation in endo/lysosomes with low pHs after internalization led to effective endo/lysosomal escape of CC-ZIFs, promoting Cas9 and sgRNA delivery to nuclei for effective knocking down of gene expression by 37% over 4 days in Chinese hamster ovary (CHO) cells. Liu et al. [99] also reported ZIFs for the delivery of CRISPR-Cas9 plasmid together with a paxillin donor template for knock-in of the paxillin gene (Fig. 2B). Paxillin is a critical protein that mediates signaling between tumor microenvironment and tumor cells and influences tumor metastasis. To produce P1-ZIF-8 (P1Z) nanostructures (P1: green fluorescent protein (GFP)-Cas9-paxillin_gRNA plasmid), plasmids were dispersed in Zn^2+^ solution and then added to 2-MIM. Additionally, plasmids were introduced in an aqueous solution containing 2-MIM, followed by the addition of Zn^2+^ solution for preparation of ZIF-8-P1 (ZP1) nanostructures. P1Z and ZP1 both showed a pH-responsive plasmid release behavior, with over 40% accumulative releasing rates in pH 5.5 and less than 15% in pH 7.4 solution. Successful endo/lysosomal escape of P1Z and ZP1 ensured the delivery of plasmids to nuclei. The paxillin gene was correctly inserted in U2OS human osteosarcoma epithelial cells.

**Fig. 3:**
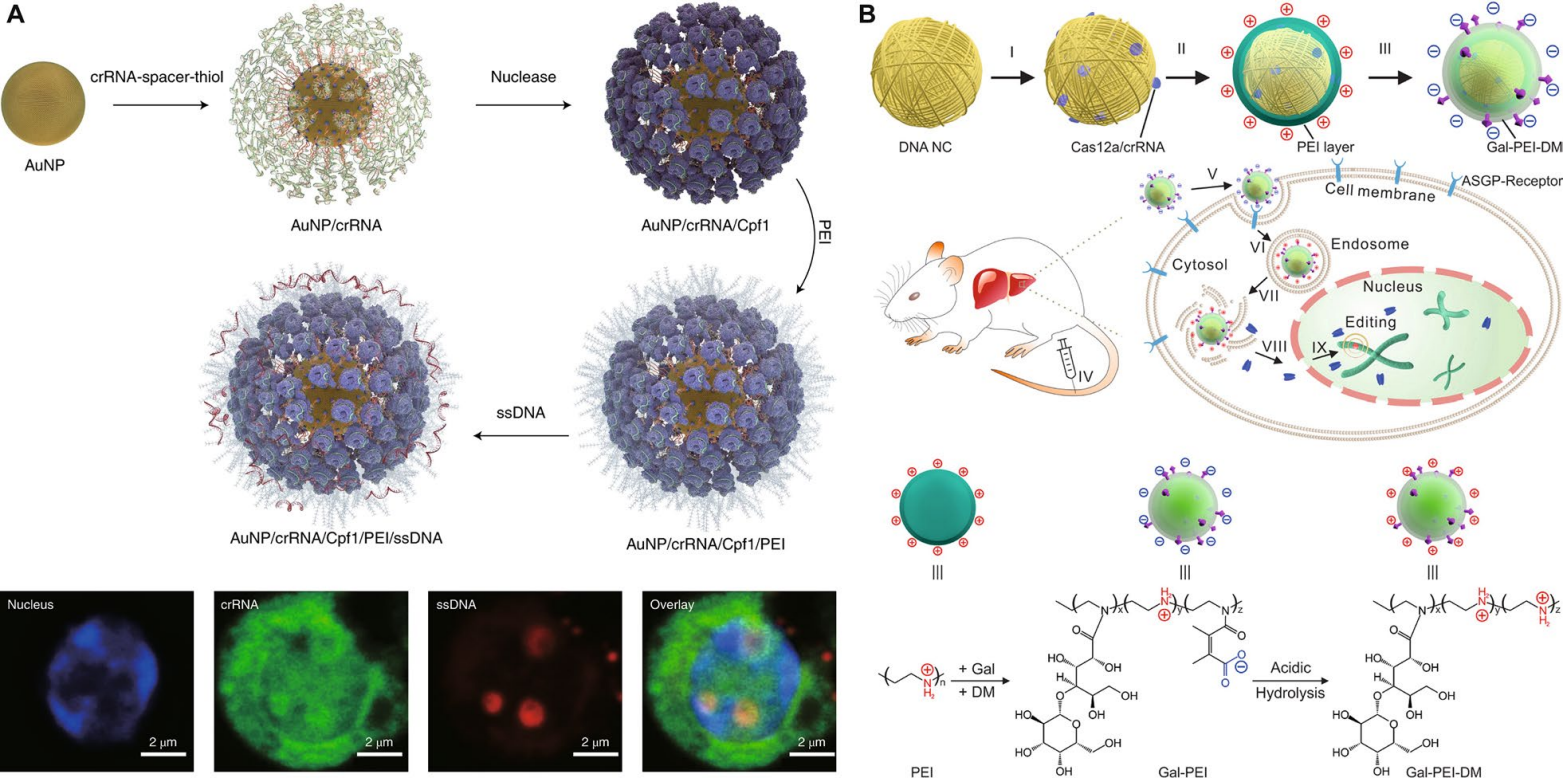
PEI-based pH-responsive CRISPR-Cas9 delivery nanoformulations. **A** Preparation of AuNP/crRNA/Cpf1/PEI/ssDNA, and laser scanning confocal microscope images of crRNA and ssDNA colocalization in a primary human CD34^+^ HSPC after incubation with AuNP/CRISPR. Reproduced with permission [106, 109]. **B** Design of the DNA NC–based charge reversal assembly for CRISPR-Cas12a delivery: preparation of the assembly of Cas12a/crRNA/NC/PEI/Gal-PEI-DM, and the chemical structure of Gal-PEI-DM and mechanism for acidic environment triggered charge reversal. Reproduced with permission [108]

Poly(ethylenimine) (PEI) is a commonly-used cationic polymer for triggering endo/lysosomal escape through the proton sponge effect, owing to the abundant amino groups in PEI that can be protonated in endo/lysosomes as well as excellent proton buffering capacities under various acidic conditions, endowing PEI a pH-responsiveness behavior [97, 100–105]. Shahbazi et al. [106] designed a gold nanoparticle (AuNP)-based nanoformulation using a layer-by-layer self-assembly strategy. crRNA-18 spacer-SH, the guide RNA, was conjugated on AuNPs, followed by the nuclease attachment to the 5ʹ handle of crRNA and then PEI coating (Fig. 3A). Donor DNA was also attached to the surface of NPs to achieve “homology-directed repair template” (HDT). PEI induced facilitated escape of nanoformulations from endo/lysosomes in hard-to-transfect primary hematopoietic stem and progenitor cells (HSPCs). Notably, the concentration of suspended AuNP cores at 10 µg ml^− 1^ demonstrated the highest editing and HDR rates in primary HSPCs, while higher concentrations led to increased cytotoxicity and lower HDR rates. Importantly, primary human CD34^+^ HSPCs were first treated with AuNP/CRISPR-HDT in vitro and then infused into sub-lethally irradiated immunodeficient (Il2r gamma^−/−^) mice. AuNP/CRISPR-HDT-treated HSPCs could allow for engrafting at higher levels than the mock (untreated) cells. In another study, Sun et al. [107] recently also established a DNA nanoclew (NC)-based layer-by-layer formulation for hepatocyte-targeted delivery of the Cas12a/crRNA RNP to regulate serum cholesterol levels (Fig. 3B). Cas12a is another type of genome editing machinery in the CRISPR-Cas9 family. PEI was applied to condensing the Cas12a/crRNA/nanoclew core, which was further coated with an anionic galactose-PEI-2,3-dimethylmaleic anhydride (DM) layer that can be charge-converged upon exposure to an acidic endo/lysosomal environment to facilitate endosome disruption and payload release. *Pcsk9* was chosen as the target gene because proprotein convertase subtilisin/kexin type 9 (PCSK9) proteins are a key regulator of serum cholesterol levels. PCSK9 is secreted by the liver and can bind with low-density lipoprotein (LDL) receptors, a key receptor that mediates endocytosis of cholesterol. Two crRNAs targeting exon2 (crRNA-exon2) and exon3 (crRNA-exon3) were designed, and corresponding Cas12a/crRNA-exon2/NC-exon2/PEI/Gal-PEI-DM and Cas12a/crRNA-exon3/NC-exon3/PEI/Gal-PEI-DM nanoparticles (NPs) were prepared, inducing ~ 75% and ~ 44% indel formation in vitro in 3T3-L1 cells (the fibroblast isolated from the embryos of mice), respectively. ~48% disruption of *Pcsk9* gene was achieved in vivo after Cas12a/crRNA-exon3/NC-exon3/PEI/Gal-PEI-DM treatment, decreasing the expression of PCSK9 enzymes and ~ 45% of cholesterol reduction. The same group [108] loaded Cas9/sgRNA RNP onto a yarn-like DNA NC, and integrated PEI as an out-layer coating for endo/lysosomal escape for enhanced RNP delivery into U2OS.EGFP (EGFP: enhanced green fluorescent protein) tumor cells.

**Fig. 4:**
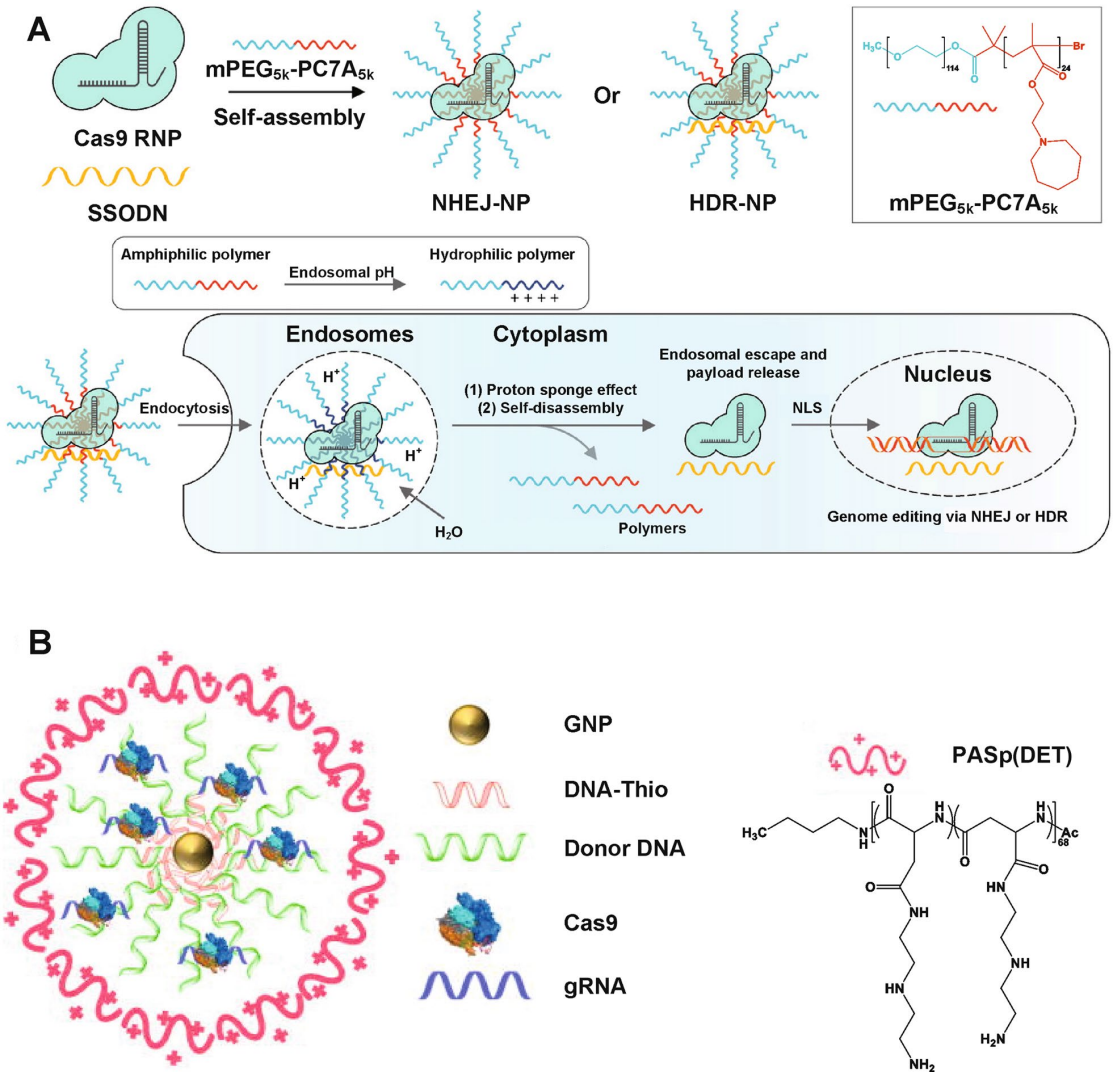
Other cationic polymer-based pH-responsive CRISPR-Cas9 delivery nanoformulations. **A** Design and preparation of NPs by pH-sensitive mPEG-PC7A polymer via self-assembly, loading either Cas9 RNP alone or both Cas9 RNP and single-strand oligonucleotides (ssODN, a donor DNA), as well as behaviors of NPs including endocytosis, endosomal escape, payload releasing in cytoplasm and delivery into nucleus. Reproduced with permission [110]. **B** Structure of CRISPR-Gold. Reproduced with permission [111]

Other pH-responsive cationic polymers with excellent proton buffering capabilities have also been used for CRISPR-Cas9 delivery. Xie et al. [110] synthesized a diblock pH-responsive amphiphilic copolymer, namely methoxy-poly(ethylene glycol)-b-poly(2-(azepan-1-yl)ethyl methacrylate) (mPEG-PC7A), to load Cas9 RNPs for gene disruption via nonhomologous end joining (NHEJ-NPs) or to load Cas9 RNPs with donor DNA templates (ssODN) for gene correction via homology-directed repair (HDR-NPs) (Fig. 4A). After endocytosis, mPEG-PC7A polymers were protonated in the acidic endo/lysosomal environment, facilitating the endosomal escape of the payloads *via* the proton sponge effect and disassembly of NPs, therefore, releasing the payloads. Intravenously, intratracheally, and intramuscularly injected NHEJ-NP induced efficient gene editing in the liver, lung, and skeletal muscle of Ai14 mice, respectively. Importantly, intramuscularly injected HDR-NPs achieved muscle strength recovery in a Duchenne muscular dystrophy (DMD) mouse model. Lee et al. [111] prepared a gold NP-based vehicle to deliver Cas9 RNPs and donor DNAs (denoted as CRISPR–Gold, Fig. 4B). DNA-thiol attached onto gold NPs can be complexed with donor DNA, Cas9 RNP, and the endosomal disruptive pH-responsive polymer poly(N-(N-(2-aminoethyl)-2-aminoethyl) aspartamide) (Pasp(DET)), *via* ​either hybridization or electrostatic interactions. After endocytosis, the Pasp(DET) polymer triggered endosomal disruption via the proton sponge effect. CRISPR–Gold induced homology-directed repair (HDR) in the dystrophin gene for DMD therapy, leading to 5.4% of the dystrophin gene in *mdx* mice corrected back to the wild-type gene.

**Fig. 5:**
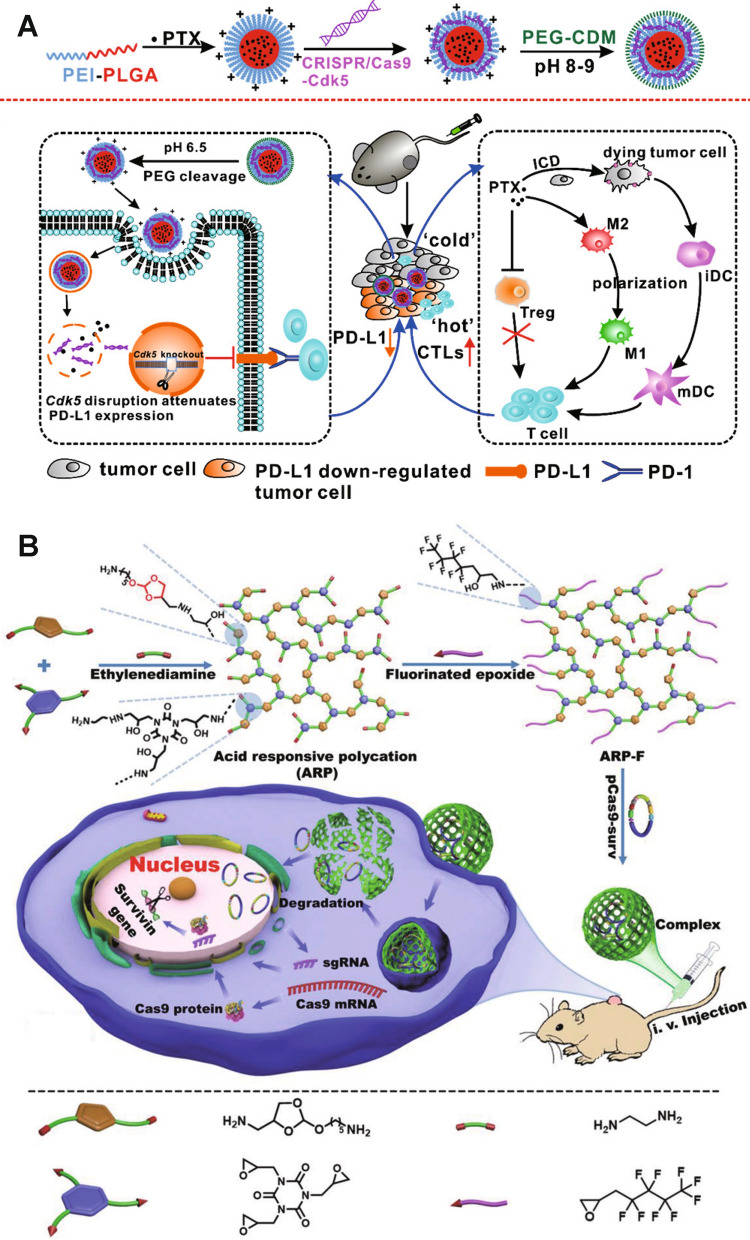
pH-responsive CRISPR-Cas9 delivery nanoformulations with pH-sensitive linkers. **A** Schematic illustrations of pH-responsive nanoparticles for PTX and CRISPR-Cas9-Cdk5 delivery and pH-responsive nanoparticle-mediated gene disruption and immunochemotherapy. Reprinted (adapted) with permission from [115]. Copyright 2022 American Chemical Society. **B** Schematic illustration of the preparation of fluorinated ARP-F and its resultant plasmid (e.g., pCas9-surv) delivery process. Reproduced with permission [116]

pH-sensitive chemical bonds, such as hydrazone bonds, ortho-ester bonds and amide bonds, that can be easily cleaved in acidic environments and lead to dissociation of NPs, have also been integrated into nanoformulations for pH-responsive CRISPR-Cas9 delivery [112–114]. Tu et al. [115] co-delivered CRISPR-Cas9-Cdk5 plasmid and paclitaxel in a nanovehicle consisting of a poly(ethyleneimine)-poly(lactic-*co*-glycolic acid) (PEI-PLGA) core and a poly(ethylene glycol) (PEG) shell, two of which were linked *via* an acid cleavable amide bond (Fig. 5A). Paclitaxel and CRISPR-Cas9-Cdk5 plasmid were loaded in the PLGA and PEI-formed region, respectively. Paclitaxel in the PLGA inner core was released more rapidly in lower pH environments (pH 6.4 and 5.5) than in the solution with neutral pH. CRISPR-Cas9-Cdk5 plasmid induced downregulation of programmed death-ligand 1 (PD-L1) expression (an immunosuppressive factor) on tumor cells, which boosted the anti-tumor immune responses. NPs with both CRISPR-Cas9-Cdk5 plasmid and paclitaxel showed superior tumor inhibition in both melanoma and colorectal cancer mouse models. Qi et al. [116] synthesized a fluorinated acid-responsive polycation (ARP-F) containing abundant pH-responsive ortho-ester linkages for Cas9 delivery (Fig. 5B). Positively-charged ARP-F could absorb negatively charged pCas9-surv, a Cas9 plasmid that targets and knocks out the survivin (an apoptosis inhibitor overexpressed by cancer cells) gene, to form stable nanocomplexes through electrostatic interactions. Ortho-ester linkages were hydrolyzed in acidic endo/lysosomes after internalization of nanocomplexes by tumor cells to release pCas9-surv. pCas9-surv-inducing survivin downregulation significantly delayed tumor growth compared with control groups. Furthermore, the anti-tumor efficacy can be further enhanced when combined with temozolomide, an anti-cancer drug. Liu et al. [117] prepared a multistage delivery NP (MDNP) for genome-regulating-mediated cancer therapy. MDNPs were formed by a cationic polyplex core consisting of CRISPR-dCas9 plasmid DNA and phenylboronic acid-modified polyethyleneimine (PEI-PBA), and a negatively-charged shell composed of 2,3‐dimethylmaleic anhydride (DMMA)‐modified poly(ethylene glycol)‐*b*‐polylysine (mPEG113‐b‐Plys100/DMMA). The acidic tumor microenvironment induced the decomposition of DMMA groups, thereby conversing anionic outlayer mPEG113‐b‐Plys100/DMMA polymers into cationic polymers. The separation of shell led to the exposure of cationic polyplex core, which enhanced intratumoral accumulation and internalization of NPs. PEI helped NPs’ endo/lysosomal escape, and the released payload dCas9-miR-524 intracellularly upregulated miR-524 in MDA-MB-231 breast cancer cells. miR-524 is suppressed in many types of cancer cells, and its overexpression can restrain the proliferation and migration of cancer cells, leading to suppressed tumor growth.

Protonation of certain chemical groups in acidic environments can induce a hydrophobicity-hydrophilicity transition or a charge reversion, which leads to the disassembly of NPs and releasing of payloads. Wang et al. [118] designed a solid lipid nanoparticle (SLN) decorated with three kinds of cell-penetrating peptides for delivering human antigen R (HuR) CRISPR-Cas9 plasmid and sgRNA (HuR/CRISPR SLN-HPR). pH-sensitive H-peptide can switch from hydrophobicity to hydrophilicity in an acidic environment (pH from 6.0 to 6.5 in the tumor microenvironment) due to the protonation of imidazolyl groups, whose conformation changes induced an open and polar structure, thereby exposing the hidden P-peptides for targeting epithelial growth factor receptor (EGFR) on human tongue squamous carcinoma SAS cells and R-peptides for targeting nuclei. The hydrophobic-to-hydrophilic transition in the acidic environment also promoted payloads release. This SLN-based CRISPR-Cas9 gene-editing system knocked out HuR, an RNA binding protein whose overexpression upregulates cancer-related transcripts of survival and resistance in various cancers, thus enhancing the antitumor efficacy of epirubicin chemotherapy. Zhang et al. [119] reported sorafenib and CRISPR-Cas9 co-loaded hollow mesoporous silica NPs that were coated with anti-epithelial cell adhesion molecules (EpCAM) aptamers as a targeting ligand and PAMAM molecules on the surface (denoted as SEHPA NPs). SEHPA NPs efficiently knocked out the EGFR gene in H22 mouse hepatocarcinoma cells and downregulated the EGFR-PI3K-Akt signaling to inhibit angiogenesis in tumor. 85% tumor inhibition was achieved in a hepatocellular carcinoma (HCC) mouse model.

Xu et al. [120] synthesized polyamidoamine (PAMAM)-based polymer for cytosolic delivery of protein, including CRISPR-Cas9 RNP. The incorporation of *N*-dibutylaminoethyl moieties strengthened the binding affinity of proteins with the hydrophobic moieties in the *N*,*N*-dialkylaminoethyl modified polymers. Furthermore, the hydrophobic-to-hydrophilic transition of tertiary amines in the *N*,*N*-dialkylaminoethyl modified polymers in acidic conditions reduced the hydrophobic–hydrophobic interactions, therefore facilitating protein release. Zhang et al. [121] reported a cationic chitosan-based nanocomplex (CLPV NPs) for co-delivery of sgVEGFR2/Cas9 plasmid and paclitaxel. Amidogen-modified chitosan molecules can be protonated at acidic endosomes or lysosomes to increase the solubility of chitosan in water, thus promoting the release of payloads. CLPV NPs exhibited genome editing efficiencies of 38.6% and 33.4% in HepG2 cells in vitro and hepatoma carcinoma in vivo, respectively. Furthermore, CLPV NPs downregulated the expression of vascular endothelial growth factor receptor 2 (VEGFR2) proteins in HepG2 cells by 60% in vitro, and suppressed tumor growth by 70% in vivo.

### Redox-responsive CRISPR-Cas9 delivery

**Fig. 6:**
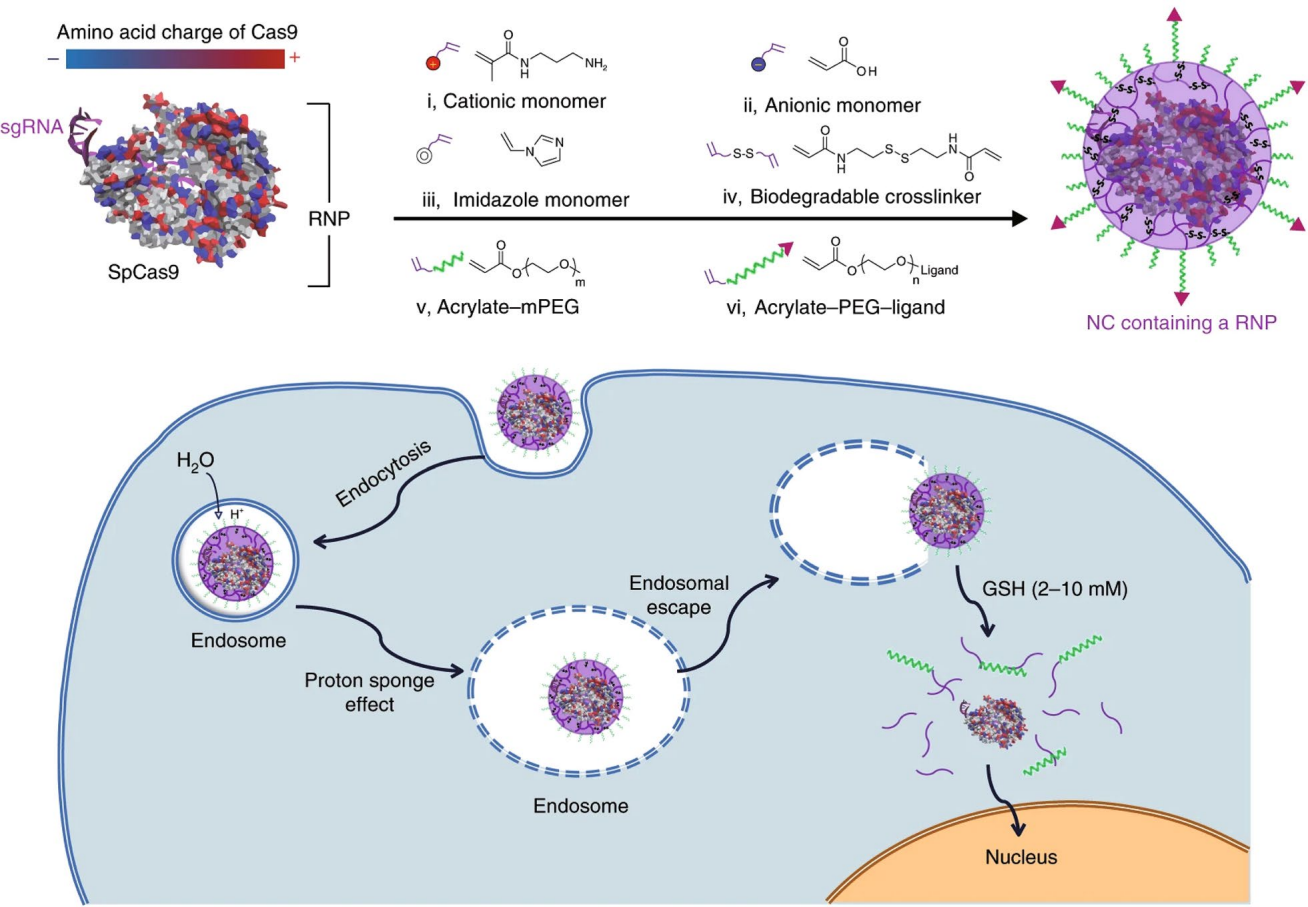
GSH-responsive CRISPR-Cas9 delivery nanoformulations. Schematic illustrations of heterogeneous surface charge of streptococcus pyogenes Cas9 (SpCas9), formation of the covalently crosslinked NC for the delivery of the Cas9 RNP complex prepared by in situ free-radical polymerization, and depiction of the proposed mechanism of the cellular uptake of NCs and the subcellular release of the RNP. Reproduced with permission [127]

It is well-documented that redox potential varies between intracellular and extracellular environments [90]. For example, a reducing agent, glutathione (GSH), is found at a level that is two to three orders of magnitude higher in the intracellular space than in the extracellular fluid [74, 122]. Hence, the integration of GSH-cleavable disulfide or diselenide bonds in the delivery systems can allow for the site-specific intracellular payload release [123–126]. Chen et al. [127] reported a GSH-degradable polymeric nanocapsule system for CRISPR-Cas9 RNP delivery (Fig. 6). RNP nanocapsule was formed by in situ crosslinking a mixture of cationic and anionic monomers using a GSH-degradable crosslinker, N,N′-bis(acryloyl)cystamine, containing a disulfide bond surrounding a Cas9 RNP. The disulfide bonds can be easily cleaved in the presence of GSH-rich cytosol, leading to the disassociation of the crosslinked polymeric shell and the release of RNP for genome editing. Encouragingly, this RNP nanocapsule system processed excellent biocompatibility and remained stable after freeze-drying. It also showed robust genome editing capabilities in multiple cells in vitro (e.g., HEK293, T cells, and stem cells) and in multiple tissues in vivo (e.g., retinal pigment epithelium tissue (RPE) and skeletal muscle in Ai14 mice), and in different gene loci (e.g., mCherry gene, APP gene, STOP-cassette gene).

**Fig. 7:**
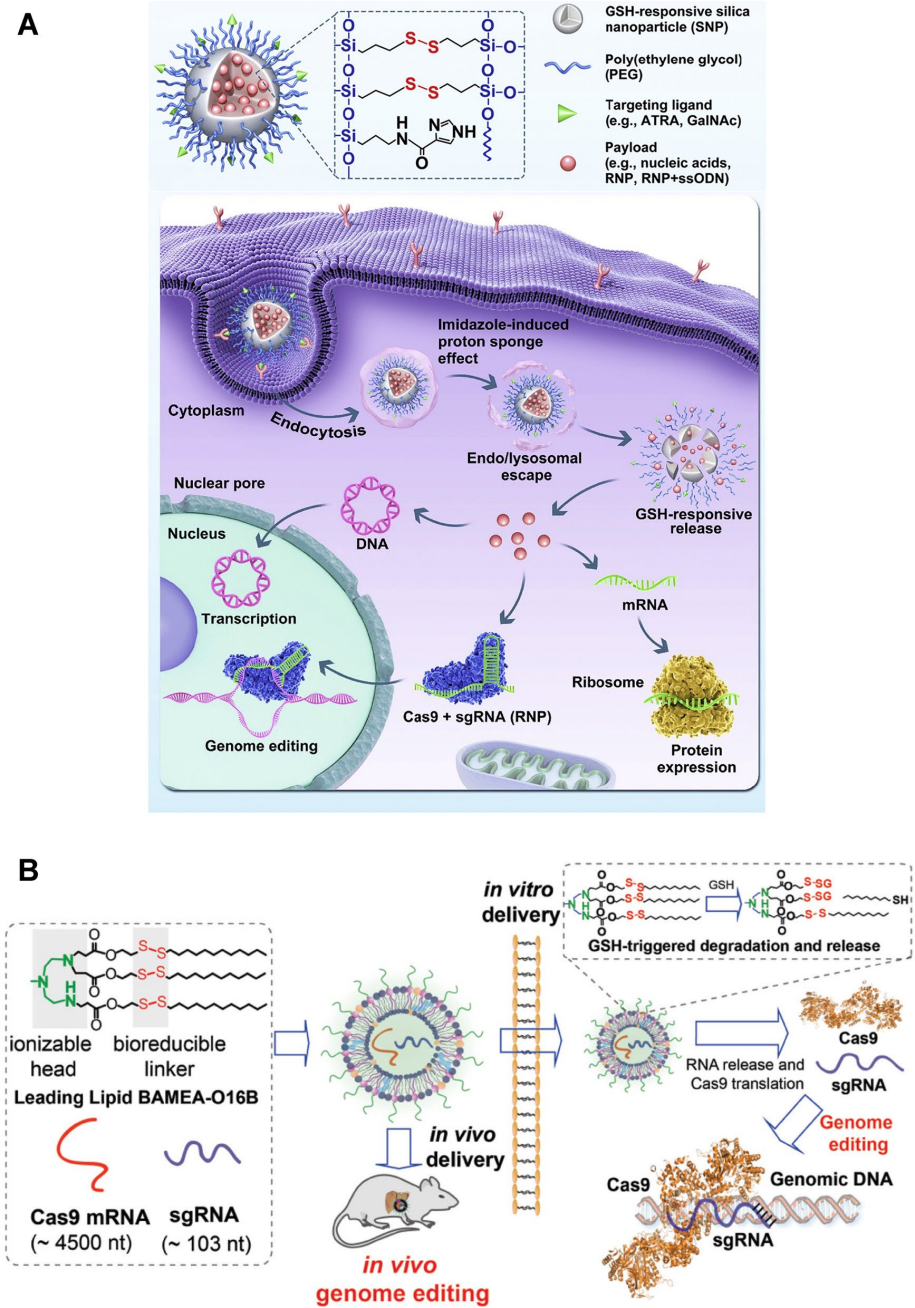
GSH-responsive CRISPR-Cas9 delivery nanoformulations. **A** Illustration of the multifunctional SNP for the delivery of nucleic acids (e.g., DNA and mRNA) and CRISPR genome editor (e.g., RNP, RNP + ssODN), and the intracellular trafficking pathways of SNP. Reproduced with permission [128]. **B** Illustration of formulating bioreducible lipid/Cas9 mRNA/sgRNA nanoparticle for CRISPR-Cas9 genome editing delivery in vitro and in vivo. Reproduced with permission [129]

Wang et al. [128] reported GSH-responsive silica nanoparticles (SNP) for RNP delivery (Fig. 7A). Cas9 RNP was loaded in the disulfide bond-containing SNP, and was released rapidly in the cell cytosol with high GSH concentrations. All-trans-retinoic acid (ATRA, an RPE-targeting molecule)-decorated Cas9 RNP-loaded SNP delivered RNP into RPE of Ai14 transgenic mice *via* subretinal injection. RNP could also be targetedly delivered to the liver by the N-acetylgalactosamine-conjugated SNP. Liu et al. [129] designed a GSH-responsive lipid, BAMEA-O16B, to deliver Cas9 mRNA and sgRNA (Fig. 7B). BAMEA-O16B with disulfide bond-containing hydrophobic tails could encapsulate RNA *via* electrostatic interactions. RNAs were effectively released in the GSH-rich intracellular environment. It produced 90% knockout efficiency of GFP expression of human embryonic kidney (HEK) cells and 80% decrease of mouse serum proprotein convertase subtilisin/kexin type 9 (PCSK9) level in vivo compared to untreated controls. Wang et al. [130] developed GSH-reducible nanocomplex for RNP delivery. Nanocomplex were formed by cationic poly(*N*,*N*′-bis(acryloyl)cystamine-co-triethylenetetramine) polymers (PBAP) with RNP. PBAP contained disulfide bonds and imidazolyl groups in the molecular chains, so that NPs could quickly escape from endo/lysosomes and respond to high GSH levels in tumor cell cytosol for RNP release. Similarly, Chen et al. [131] synthesized cationic block copolymer, poly(aspartic acid-(2-aminoethyl disulfide)-(4-imidazolecarboxylic acid))–poly(ethylene glycol) (P(Asp-AED-ICA)-PEG), containing disulfide bonds and imidazolyl groups to prepare nanocomplex *via* electrostatic interaction with negatively-charged Cas9 RNP. Cas9 RNP can be released in tumor cells due to the cleavage of disulfide bonds by GSH.

Qi et al. [132] synthesized a branched cationic biopolymer (LBP) containing multiple disulfide linkages and hydroxyl groups to deliver Cas9 plasmids to hepatocellular carcinoma (HCC) cells. The cleavage of disulfide bonds triggered the release of pCas9-survivin to knockout the surviving gene, a gene that is highly expressed in almost all kinds of cancers as the target for antitumor intervention. The LBP/pCas9 complex successively resulted in genome editing in HCC, where the knockout of the survivin gene led to apoptosis and proliferation inhibition of tumor cells in vitro, and the gene-editing efficiency was 26.4% in orthotopic HCC mouse models. Lu et al. [133] developed a reduction-sensitive fluorinated-Pt(IV) transfection nanoplatform (PtUTP-F) containing reducible inert Pt(IV) prodrug, fluorinated polyethyleneimine, and dCas9-CT45 plasmids. GSH could induce the collapse of PtUTP-F/dCas9-CT45 NPs and release of plasmids and cytotoxic Pt(II) drugs. Expression of CT45 induced by dCas-CT45 plasmids sensitized A2780 ovarian cancer cells to the anticancer Pt(II) drugs, inhibiting tumor growth. Wan et al. [134] reported an RNP-loaded bioreducible nanocomplex by integrating the disulfide bonds in the adamantine/CD host-guest interactions. Cas9 could tightly adhere to biguanidyl groups on the polymer surface by strong hydrogen bonding and salt bridges. GSH induced the cleavage of disulfide bonds and thus released RNP. Disruption of mutant *KRAS* gene of CRC cells by this nanocomplex led to effective inhibition of tumor growth and metastasis in vivo. Liu et al. [135] loaded axitinib, a small molecule inhibitor of tyrosine kinase, into the pores of surface-thiolated mesoporous silica nanoparticles (MSN-SH), and sealed the pores by conjugating Cas9/sgRNA RNP through disulfide bonds. The detachment of Cas9/sgRNA was facilitated in the presence of GSH in cancer cells. Programmed cell death 1 (PD-1) was knocked out in B16F10 cells to disrupt the immunosuppressive PD-1/PD-L1 pathway for enhanced cancer immunotherapy.

**Fig. 8:**
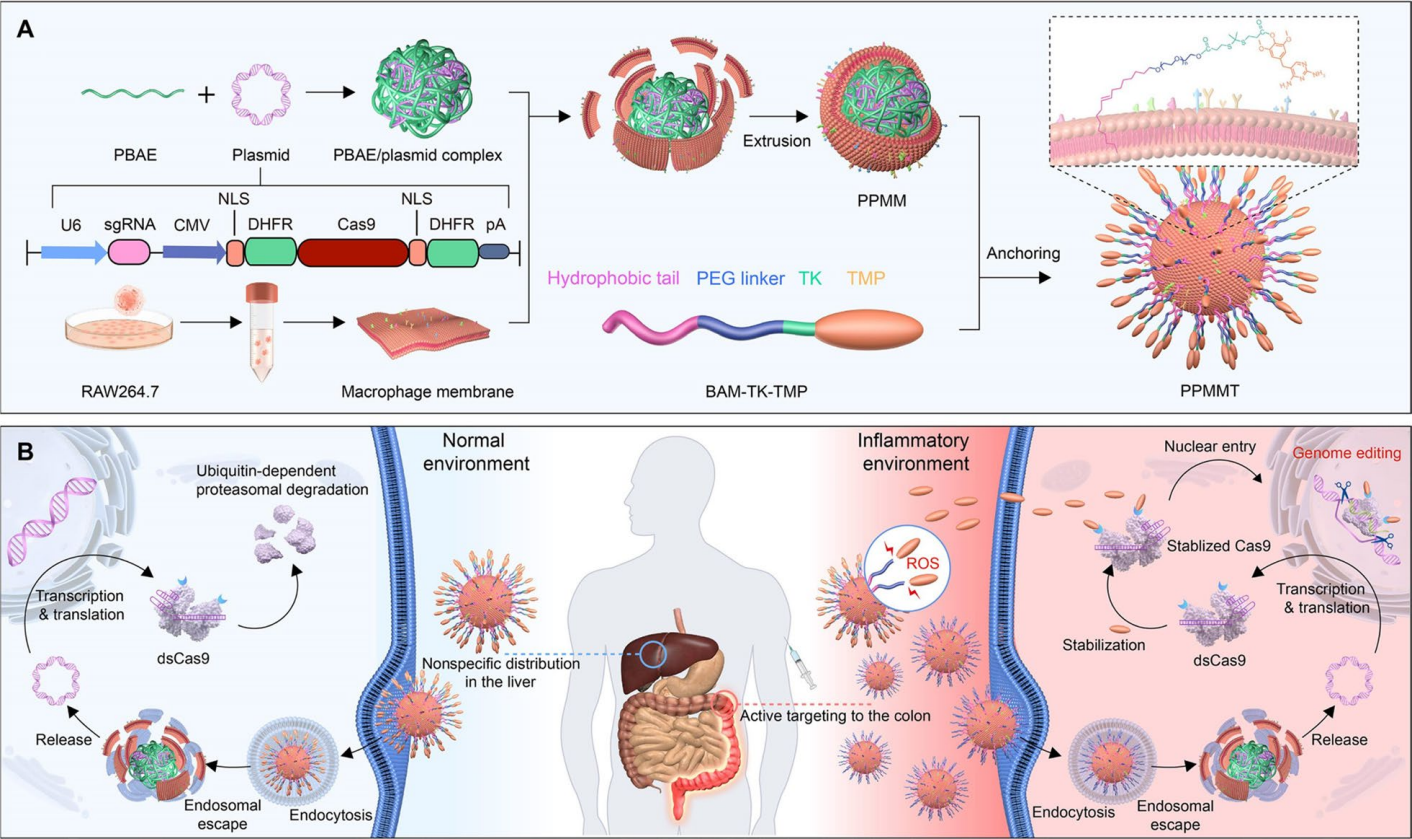
ROS-responsive CRISPR-Cas9 delivery nanoformulations. **A** Process of preparation of the PPMMT complex: design of the plasmid encoding the dsCas9 sequence with DHFR domains, complexation with the cationic polymer (PBAE), coating with MMs derived from RAW264.7 cells, and anchoring of ROS-responsive BAM-TK-TMP on the membrane of PPMM to obtain PPMMT. **B** Illustration of targeted delivery and inflammation-specific genome editing in the inflammatory colon lesion: PPMMT targeting the inflammatory colons by virtue of MMs, plasmid translation into dsCas9, dsCas9 stabilized upon the released TMP to recover the genome-editing activity, and dsCas9 degradation in a ubiquitin-dependent proteasomal pathway in the noninflammatory tissues. Reproduced with permission [143]

In addition to the aforementioned reducing intracellular conditions, elevation of reactive oxygen species (ROS) concentration (e.g., hydrogen peroxide (H_2_O_2_) and hydroxyl radicals) is also detected with distinct pathological conditions, including cancer, stroke, arteriosclerosis, and tissue injuries [136–139]. Tumor cells, for instance, are associated with a much higher ROS level than normal cells [140–142]. Yan et al. [143] reported a genome editing prodrug system (NanoProCas9) for CRISPR-Cas9 targeted delivery and conditional stabilization (Fig. 8). Plasmid DNA encoding destabilized Cas9 (dsCas9) with dihydrofolate reductase (DHFR) domains was complexed with cationic poly(*β*-amino ester) (PBAE) polymers. Subsequently, macrophage membranes (MM) were coated on the surface of PBAE/plasmid complex surface for targeted delivery of dsCas9 to inflammatory lesions. Lastly, trimethoprim (TMP), a small-molecule stabilizer for dsCas9 was conjugated to oleyl ether–modified poly(ethylene glycol) (OE-PEG) through a thioketal linker to obtain ROS-responsive BAM-TK-TMP, and the precursory molecule BAM-TK-TMP with the hydrophobic tail was anchored on the MM through lipid fusion. In a normal physiological environment, the expressed structurally unstable dsCas9 is prone to degradation by ubiquitin-dependent proteasomes. In a ROS-rich environment such as inflammatory lesions, BAM-TK-TMP was activated to release TMP that can stabilize dsCas9. This ROS-dependent NanoProCas9 system achieved efficient delivery and targeted activation of CRISPR-Cas9 in response to high ROS levels for genome editing in BALB/c mice with dextran sulfate sodium-induced colitis.

### Enzyme-responsive CRISPR-Cas9 delivery

Alteration in the expression of certain enzymes often occurs in pathological conditions, which has been exploited to achieve enzyme-responsive drug delivery at the desired target [144–147]. Most enzyme-responsive drug delivery systems utilize enzymes present in the extracellular space. For example, matrix metalloproteinases (MMPs) and hyaluronidase (HAase) are often upregulated in the tumor microenvironment [148, 149]. MMPs play important roles in microenvironment modeling and signaling pathway regulating during tumorigenesis, tumor progression, and tumor metastasis [150, 151]. High levels of hyaluronic acid (HA) in the tumor microenvironment reduce the elasticity of tumor tissues and increase interstitial fluid pressure, and are correlated with poor prognosis in many cancer [152]. Yin et al. [153] developed a supercharged polypeptide (SCP) system linked with Cas9/sgRNA through a dithiocyclopeptide linker comprising an MMP-2 sensitive sequence and an intramolecular disulfide bond. The MMP-2-sensitive sequence in the dithiocyclopeptide linker was broken down by MMP-2 overexpressed in the tumor microenvironment, leading to a tumor-specific cleavage. The remaining disulfide bond in the dithiocyclopeptide linker was then degraded by intracellular GSH and eventually released RNP. It showed 31.9% editing efficiency in HeLa cells.

**Fig. 9:**
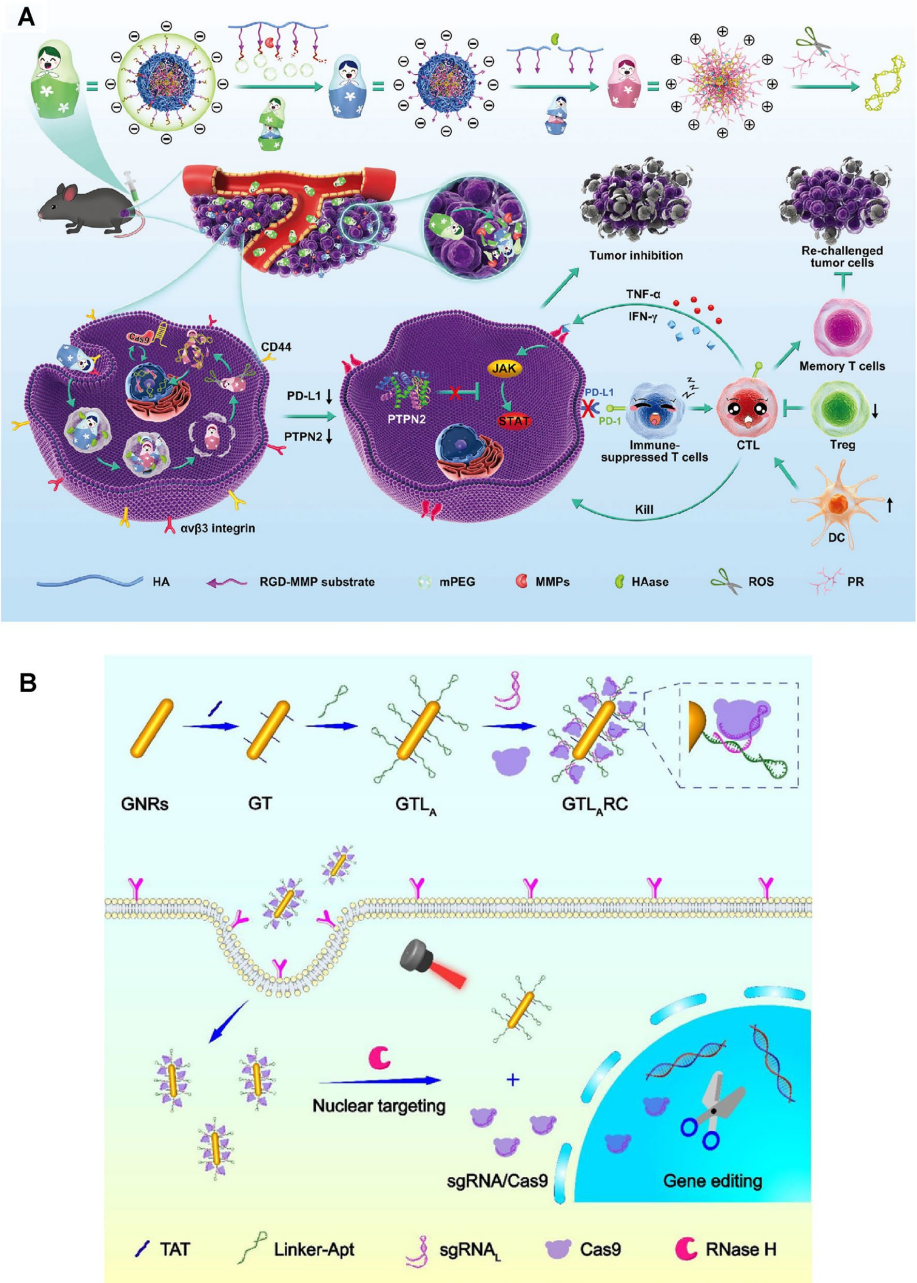
Enzyme-responsive CRISPR-Cas9 delivery nanoformulations. **A** Design and immunotherapeutic functions of PUN@Cas-PT: fabrication and the programmable unlocking process of PUN@Cas-PT in response to MMPs, HAase, and ROS, and schematic illustration of the utilization of PUN@Cas-PT for efficient multitargeted ICB therapy in vivo. Reproduced with permission [154]. **B** Schematic illustration of the design of the CRISPR-Cas9 gene-editing system loaded multifunctional nanoplatform (GTLARC: gold nanorod modified with TAT and linker-Apt to load sgRNA/Cas9 complex) and targeted gene editing and combined tumor therapy based on the multifunctional nanoplatform. Reprinted (adapted) with permission from [162]. Copyright 2022 American Chemical Society

Yang et al. [154] designed a programmable unlocking nano-matryoshka-CRISPR system (PUN@Cas-PT) that targeted PD-L1 (an immunosuppressive marker in tumor cells) and protein tyrosine phosphatase N2 (PTPN2, a negative regulator rendering tumor resistant to immunotherapy) (Fig. 9A). PUN consisted of dual MMP- and HAase-responsive corona layers and redox-responsive core, aiming to prolong the circulation time, enhance retention and internalization in tumor, and promote endo/lysosomal escape and intracellular payloads release. PUN@Cas-PT processed three stages of stimuli-responsiveness. Firstly, the MMP-RGD substrate linked with the PEG outlayer was degraded by MMP in the tumor microenvironment, exposing RGD peptides and hyaluronic acids for facilitated tumor cell recognition and internalization. HAase presented in endo/lysosomes then degraded hyaluronic acids layer to induce a negative-to-positive charge reversion, leading to endo/lysosomal escape. Finally, the core of the nanocomplex formed by ROS-sensitive polyethyleneimine derivative and CRISPR-Cas9 plasmids was disassembled in cytosol with high ROS levels, releasing CRISPR-Cas9 plasmids. Downregulation of PD-L1 and PTPN2 relieved the immunosuppressive microenvironment, amplifying anti-tumor immune responses.

Li et al. [155] established an HA-coated core-shell nanocarrier for nucleus-targeting CRISPR-Cas9 delivery. Cas9-hMTH1 pDNA was bound with fluorinated polymer (PF_33_) as the core of the nanocarrier. When delivered into tumor tissues, the HA coating was degraded by HAase, exposing the positively charged PF_33_-Cas9 core that can enhance both cell internalization and CRISPR-Cas9 delivery into the nucleus. The *MutT Homolog1* (MTH1, a pyrophosphate overexpressed in tumor cells that prevent cells from DNA damage and apoptosis [156]) gene was effectively knocked out (~ 44%) in SKOV3 human ovarian cancer cells and tumor growth was significantly inhibited in vivo.

Ribonuclease H (Rnase H) is an endoribonuclease that widely exists in mammalian cells, saccharomyces, prokaryotes, and virus particles [157–160]. Rnase H is able to specifically hydrolyze RNA segments in DNA–RNA hybrid chains, however, it cannot digest single- or double-stranded DNA or RNA alone [161]. Taking advantage of this specificity, Tang et al. [162] prepared a gold nanorod-based Cas9/sgRNA-DNA hybrid nanocarrier for Rnase H-responsive genome editing. The gold nanorods were functionalized with a tailored DNA linker that can complementarily bind with 3’ terminal-extended sgRNA in the RNP. Additionally, the DNA linker was also tailored with cell-type-specific aptamers for active-cell targeting (Fig. 9B). After cellular internalization, intracellular Rnase H specifically digested the RNA moiety in the RNA-DNA hybrids to release Cas9/sgRNA RNP, ultimately leading to 20% genomic modification of polo-like kinase 1 (PLK-1) in MCF-7 cells.

### ATP-responsive CRISPR-Cas9 delivery

**Fig. 10:**
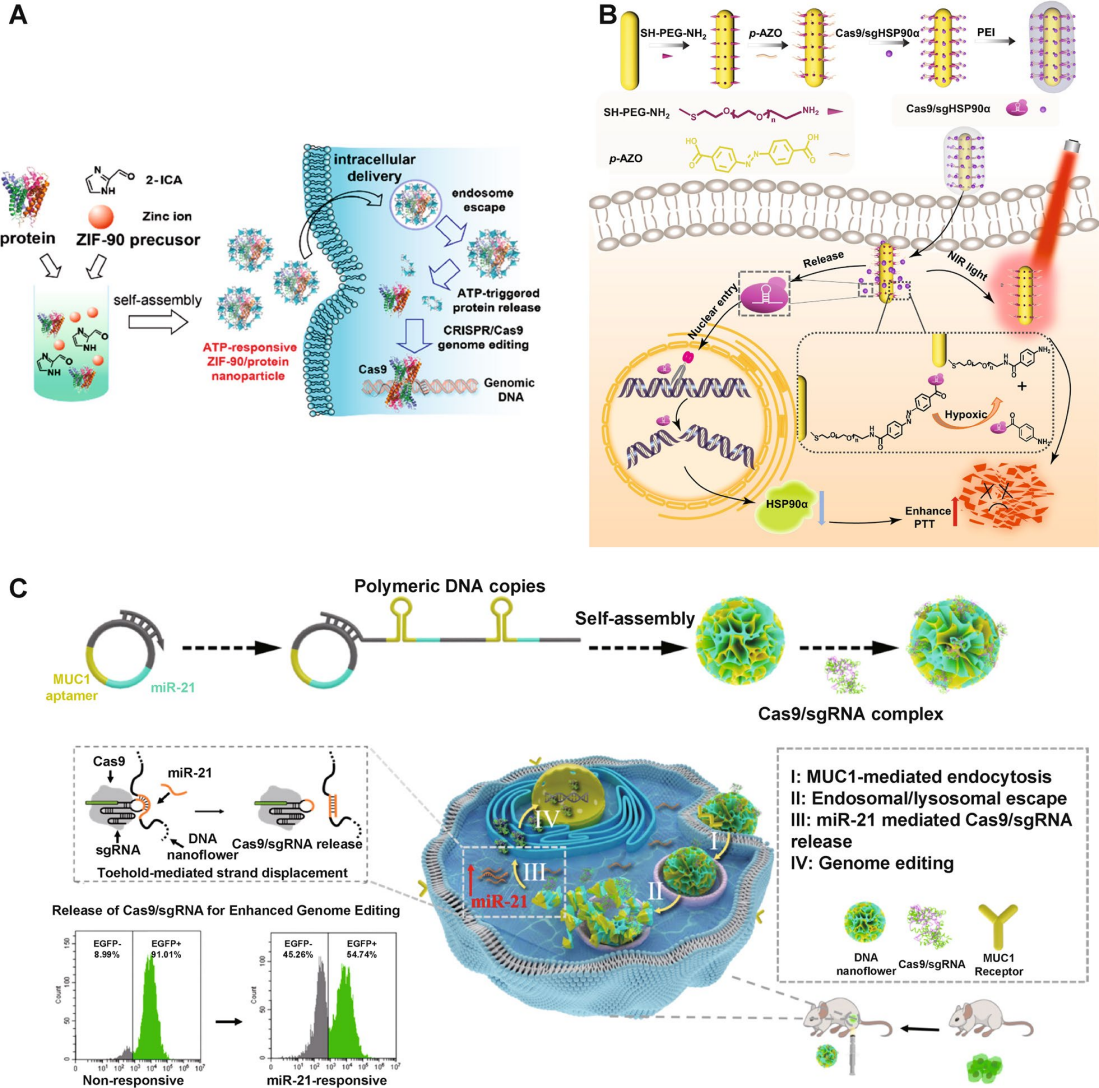
**A** ATP-responsive CRISPR-Cas9 delivery nanoformulations. Schematic illustration of the self-assembly of ZIF-90/protein nanoparticle and ATP-triggered protein release from ZIF-90 nanoparticle inside cells. Reprinted (adapted) with permission from [166]. Copyright 2022 American Chemical Society. **B** Hypoxia-responsive CRISPR-Cas9 delivery nanoformulations. Design of the hypoxia-responsive CRISPR-Cas9 system to reduce tumor thermal tolerance, and hypoxia-triggered delivery of Cas9-sgHSP90*α* to the nucleus for gene editing and mild-photothermal therapy carried out with NIR light. Reproduced with permission [169]. **C** MicroRNA-responsive CRISPR-Cas9 delivery nanoformulations. Schematic diagram of microRNA-responsive DNA nanoflower for release of Cas9/sgRNA and enhanced genome editing. Reproduced with permission [176]

ATP is the primary energy source for living cells. ATP concentrations in tumor sites (100–500 µM) are typically 1000 times higher than those in healthy tissues (10–100 nM), offering possibilities to engineer ATP-responsive nanoformulations for CRISPR-Cas9 delivery and activation [73, 163–165]. Yang et al. [166] reported a zeolitic imidazole framework-90/Cas9 (ZIF-90/Cas9) NP that was self-assembled from imidazole-2-carboxaldehyde and Zn^2+^ with Cas9-EGFP proteins (Fig. 10A). ATP induced degradation of the ZIF-90 framework due to its competitive coordination with Zn^2+^, leading to an ATP-responsible release of Cas9. ZIF-90/Cas9 NPs led to 40% knockout of GFP in HeLa-GFP cells.

### Hypoxia-responsive CRISPR-Cas9 delivery

Hypoxia is a hallmark pathological feature in many diseases, particularly in solid tumors [167]. Rapid proliferation of tumor cells and the dysfunction of blood vessels in tumor lead to a decreased oxygen supply, forming a hypoxic tumor microenvironment [168]. Li et al. [169] established hypoxia-responsive gold nanorods (AuNRs)-based CRISPR-Cas9 nanocomplex for *heat shock protein 90* (Hsp90*α*) gene knockout (Fig. 10B). Cas9/sgRNA was linked to AuNRs through a hypoxia-sensitive linker, namely azobenzene-4,4′-dicarboxylic acid. As the imbalance of cellular redox states in the hypoxic microenvironment of tumor cells contributed to an increase in reducing stress, the azobenzene-4,4′-dicarboxylic acid linker could be reduced, thus releasing Cas9/sgRNA from AuNRs. Successful *Hsp90α* gene knockout sensitized A549 tumor cells to hyperthermia by AuNPs photothermal conversion.

### MicroRNA-responsive CRISPR-Cas9 delivery

MicroRNAs (miRNAs) are non-coding, single-stranded RNAs consisting of ~ 22 nucleotides and can regulate multifarious cellular processes, such as proliferation, cell growth, and differentiation [170]. Dysregulated miRNA expression has been found closely related to tumorigenesis [171]. Several types of miRNAs (e.g., oncomiRs in cancer) are specifically expressed in certain cancer cell lines, making miRNA a possible internal trigger for bioresponsive CRISPR-Cas9 genome editing [172, 173]. Hirosawa et al. [174, 175] integrated the miRNA-mediated switch technology in the CRISPR-Cas9 system to establish miR-Cas9-OFF and miR-Cas9-ON platforms, realizing miRNA-responsive Cas9 activation in cells. This on-demand Cas9 activation and deactivation behavior has been achieved in HeLa cells in *vitro*. Shi et al. [176] prepared DNA nanoflowers (DNFs) containing miR-21 binding sequences (Fig. 10C). Cas9/sgRNA with an extended sequence that was 7 nt shorter than miR-21 was able to be loaded on DNFs by sequence hybridization. In the cytoplasm, endogenous miR-21 can replace Cas9/sgRNA from DNF by toehold-mediated sequence displacement to release CRISPR-Cas9 RNP, leading to efficient genome editing in Hela cells both in vitro and in vivo.

Representative examples of internal-stimuli nanoformulations for CRISPR-Cas9 delivery are summarized in Table 1. Generally, these nanoformulations rely on intrinsic biological triggers to control the behavior or functions of CRISPR-Cas9. While many advantages of internal-stimuli nanoformulations for controllable CRISPR-Cas9 genome editing have been detailed above, due to the complicated and dynamic (patho)physiological environment in the body, the targeting and release of loaded CRISPR-Cas9 could be less controllable, leading to potential off-target effects and side effects [177, 178]. Additionally, most of these intrinsic signals are not uniquely present in the diseased cells or cells of interest, but rather present in different levels; therefore, engineering materials that can sense subtle changes of these internal stimuli could greatly benefit the precision in genome editing.

**Table 1:** Representative internal-stimuli responsive nanoformulations for CRISPR-Cas9 delivery

Stimulus	NP composition	CRISPR-Cas9 format	Target gene locus	Applications	Administration route	Refs.
pH	Cationic block copolymer P(Asp-AED-ICA)–PEG, with imidazole residues and disulfide bonds	RNP	EGFP; mCherry	In vitro (mCherry-expressing HEK 293 human embryonic kidney cells)	-	[131]
pH	Zeolitic imidazole frameworks (ZIFs)-8 co-encapsulating Cas9 protein and sgRNA	RNP	EGFP	In vitro (Chinese hamster ovary (CHO) cells)	-	[98]
pH	ZIF-8 encapsulating plasmid	Plasmid	EGFP-tagged paxillin	In vitro (U2OS human bone osteosarcoma epithelial cells)	-	[99]
pH	AuNP-based CRISPR nanoformulation	RNP	Chemokine receptor 5 (CCR5); gamma (γ)-globin	In vivo (NOD.Cg-Prkdc^scid^Il2rg^tm1Wjl^/Szj (Il2r gamma^−/−^) mice)	Intrahepatic injection of CD34^+^ cells treated by AuNP/CRISPR	[106]
pH	Cas12a/crRNA RNP-loaded DNA nanoclew with a coating of PEI and Gal-PEI-DM layers	RNP	EGFP; PCSK9	In vivo (C57BL/6 mice)	Intravenous injection	[108]
pH	pH-responsive mPEG-PC7A amphiphilic copolymer self-assembly	RNP	GFP; BFP; tdTomato; dystrophin	In vivo (B6.Cg-*Gt(ROSA)26Sor*^*tm14(CAG−tdTomato)Hze*^/J mice and C57BL/10ScSn-*Dmd*^*mdx*^/J)	Intravenous injection; intramuscular injection; and intratracheal injection	[110]
pH	DNA-conjugated gold nanoparticles complexed with PAsp(DET)	RNP	BFP; GFP; CXCR4; tdTomato; dystrophin	In vivo (Ai9 mice, C57BL/10ScSn (wild-type) mice, and C57BL/10ScSn-*Dmdmdx*/J (mdx) mice)	Intramuscular injection	[111]
pH	NP with a cationic PEI–PLGA core and a PEG corona	Plasmid	GFP; cyclin-dependent kinase 5 (Cdk5)	In vivo (B16F10 melanoma in C57BL/6 mice; CT26 colorectal carcinoma in BALB/c mice)	Intravenous injection	[115]
pH	Fluorinated acid-labile branched hydroxyl-rich polycation	Plasmid	Renilla luciferase; EGFP; survivin	In vivo (A549 epithelial carcinoma in BALB/c mice)	Intravenous injection	[116]
GSH	Nanocapsule with a GSH-cleavable covalently crosslinked polymer coating	RNP	mCherry; tdTomato; a stop cassette before tdTomato (Ai14)	In vivo (Ai14 mice)	Subretinal injection; intramuscular injection	[127]
GSH	Silica NPs integrated with disulfide bond-containing crosslinker	mRNA, or RNP	GFP; BFP; RFP; tdTomato	In vivo (Ai14 mice)	Subretinal injection; intravenous injection	[128]
GSH	Leading lipid BAMEA-O16B	mRNA/sgRNA	GFP; RFP; PCSK9	In vivo (C57BL/6 mice)	Intravenous injection	[129]
ROS	Cationic poly(*β*-amino ester) with macrophage membrane coating and precursory molecule BAM-TK-TMP anchoring	Plasmid	EGFP; prolyl hydroxylase domain 2 (PHD2)	In vivo (BALB/c mice with colitis)	Intravenous injection	[143]
MMP/HAase/ROS	Programmable unlocking nano-matryoshka-CRISPR system with ROS-sensitive PEI derivative core and coated RGD-MMP substrate, hyaluronic acid, and PEG	Plasmid	EGFP; PD-L1; PTPN2	In vivo (B16F10 melanoma in C57BL/6 mice, A375 melanoma in BALB/c nude mice)	Intravenous injection	[154]
Ribonuclease H	Gold nanorod modified with nuclear targeting peptide and cell-type-specific aptamer	RNP	EGFP; PLK1	In vitro (MCF-7 breast cancer cells)	-	[162]
ATP	ZIF-90/protein nanoparticles by self-assembly of imidazole-2-carboxaldehyde and Zn^2+^ with protein	RNP	GFP	In vitro (HeLa cervical carcinoma cells)	-	[166]
Hypoxia	Gold nanorods conjugated with azobenzene-4,4′-dicarboxylic acid linker	RNP	EGFP; Hsp90*α*	In vivo (A549 epithelial carcinoma in nude mice)	Intravenous injection	[169]
MicroRNA	DNA nanoflowers with multiple replicates of MUC1 aptamers and miR-21 binding sequences	RNP	EGFP	In vivo (HeLa cervical carcinoma in BALB/c mice)	Intratumoral injection	[176]

### External stimuli-responsive nanoformulations for CRISPR-Cas9 delivery

External stimuli-responsive CRISPR-Cas9 delivery systems offer controlled genome editing with high spatial and temporal precision. Common external stimuli explored for this type of study include light, ultrasound and magnetism. This section will highlight these external stimuli-responsive CRISPR-Cas9 genome editing.

### Light-responsive CRISPR-Cas9 delivery

Controlled delivery and activation of gene editing systems by light offer a non-invasive, spatial and temporal solution [179]. Upon light applications, materials can undergo several subsequent physical or chemical changes, including light-induced photothermal effects from photothermal agents, light-induced generation of ROS from photosensitizing substances, and light-induced photon upconversion of upconverting luminescent materials [180–182]. These unique behaviors have been recently exploited for CRISPR-Cas9 delivery and activation. Of note, near-infrared (NIR) light (> 800 nm) is a preferred light source since it has a higher penetration depth (up to 3.2 cm) compared to ultraviolet or visible lights (< 1 mm) [179, 183, 184].

First, photothermal conversion is a commonly utilized light-triggered phenomenon in biomedical applications, such as photothermal therapy [185, 186]. Photothermal agents, including organic (e.g., indocyanine green and polydopamine) and inorganic ones (e.g., gold NPs, CuS NPs and graphene), can convert light into heat energy, leading to temperature increases [187–192]. Notably, it has been validated that photothermal effects can also facilitate endo/lysosomal escape and cleavage of thermal-sensitive chemical bonds to achieve on-demand payload release and activation [193, 194]. Wang et al. [195] reported an AuNP-based photothermally controlled CRISPR-Cas9 delivery system. Cas9-sgPlk-1 plasmids were condensed on the positively charged cell-penetration peptide-functionalized AuNPs via electrostatic attraction to form AuNPs/CP. AuNPs/CP was further coated with a lipid/lipid-PEG layer to obtain lipid-encapsulated AuNPs/CP (LACP). Under light irradiation (514 nm), AuNPs produced hot electrons that can cleave Au-S bonds, leading to the release of peptides and Cas9-sgPlk-1 plasmids from the AuNPs. LACP was intratumorally injected into an A357 melanoma mouse model and was irradiated by a 514 nm light, leading to the knockout of the *Plk-1* gene. Plk-1 was overexpressed in tumor cells, disruption of which can lead to tumor apoptosis. Yin et al. [196] loaded Cas9/sgRNA onto the surface of PEI-decorated silicene nanosheets by physical adsorption and π-stacking, and obtained a silicene-Cas9 gene-editing nanosystem. Silicene is an emerging 2D allotrope of silicon with efficient photothermal-conversion efficiency, high drug-loading capacity, and desirable biocompatibility and biodegradability. The periodic atomic grooves on the surface of 2D silicene can work as abundant anchoring sites for loading proteins and RNA. The photonic hyperthermia effect triggered under NIR-II light (1064 nm) contributed to the rapid endo/lysosomal escape of NPs and release of RNP. *TXNDC5* gene weakens the PTT efficacy through protein processing in the endoplasmic reticulum signaling pathway; as a result, Cas9/sgRNA RNP knocked down the *TXNDC5* gene, amplifying the photothermal hyperthermia effect against tumor. Chen et al. [197] reported an NIR-triggered CuS-RNP/DOX@PEI nanoplatform for combined gene, photothermal and chemotherapy. CuS NPs core was linked with thiol-modified DNA fragments, on which Cas9/sgRNA was bound *via* the base complementary pairing principle and in which doxorubicin was inserted. CuS NPs, as a photothermal agent, transformed NIR light (808 nm) into heat that accelerated the breaking of hybridization of DNA fragments and sgRNA, thus releasing doxorubicin and Cas9/sgRNA RNP. RNP targeting the Hsp90*α* gene effectively downregulated expression of Hsp90*α*, a subunit of heat shock protein 90 (Hsp90), to reduce the thermal tolerance and metastasis capabilities of tumor cells. Meanwhile, the CuS NP-induced thermal effect can also kill tumor cells. In the A375 melanoma mouse model, the combination of light-triggered genome editing, photothermal therapy and DOX chemotherapy significantly suppressed tumor growth.

**Fig. 11:**
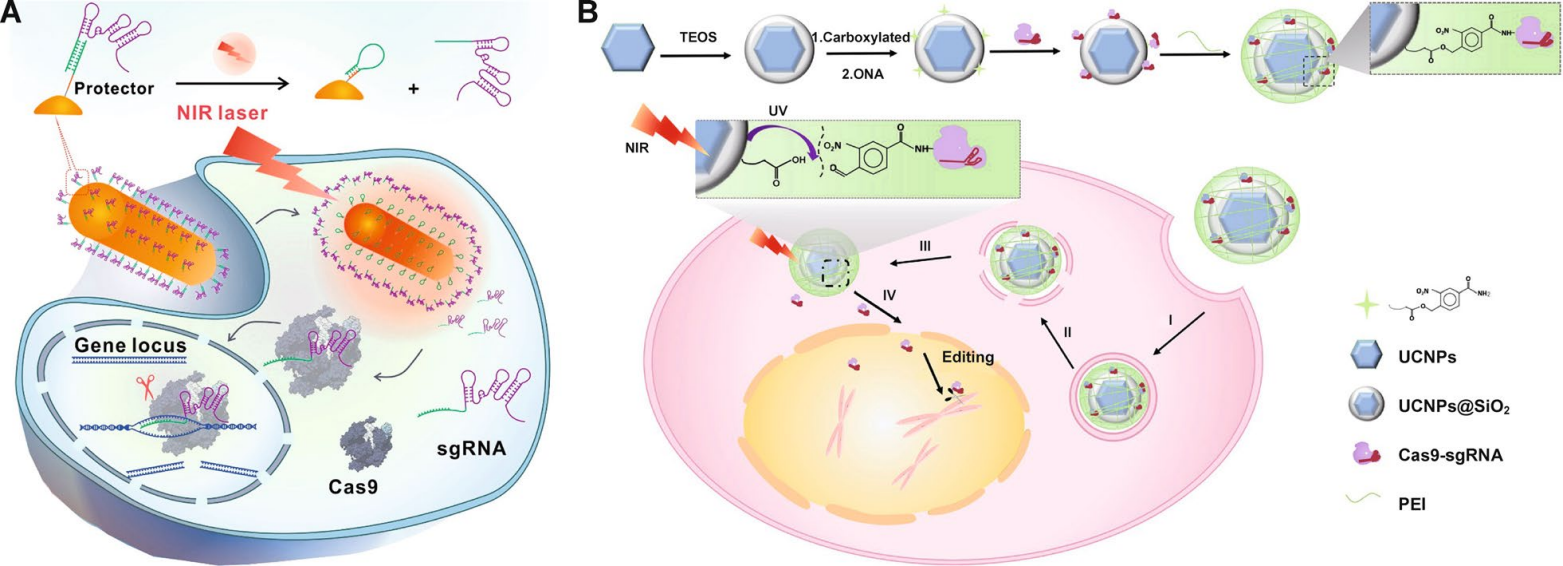
Light-responsive CRISPR-Cas9 delivery nanoformulations. **A** Delivery and intracellular activation of a CRISPR-Cas9 genome-editing nanomachine: sgRNA hybridization to a protector DNA, heat-activating sgRNA releasing, formation of hairpin structure of the protector and sgRNA releasing in the cells. Reprinted (adapted) with permission from [199]. Copyright 2022 American Chemical Society. **B** Preparation of UCNPs-Cas9@PEI and NIR-triggered delivery of Cas9-sgRNA to the nucleus of the cell for gene editing: attachment to the cell membrane, endocytosis, endosome escape, releasing from particles and stepping into the nucleus, and searching for the target DNA locus and initiate the DNA double-strand break for genome editing. Reproduced with permission [210]

Li et al. [198] utilized a semiconducting polymer (SP) functionalized with fluorinated polyethylenimine (PF) for delivering CRISPR-Cas9 plasmids and dexamethasone (Dex). Cas9 plasmids were bound to PF polymers through electrostatic and supramolecular interactions, and Dex, a molecule that can with the nuclear glucocorticoid receptor to dilate the nuclear pores, was encapsulated in the hydrophobic core of the NPs. The irradiation of NIR-II (950–1700 nm) activated SP and induced heat-mediated endo/lysosomal escape of NPs and NPs disruption to release payloads for potential site-specific precise genome editing. Additionally, the integration of Dex in this system increased the nuclear translocation of Cas9 plasmid for enhanced genome-editing efficiency. Peng et al. [199] designed gold nanorods modified with protector DNA strands for sgRNA delivery (Fig. 11A). The protector DNA was hybrid with sgRNA for sgRNA loading. After intracellular delivery into A549-GFP/Cas9 cells stably expressing GFP and Cas9 proteins, the gold nanorods under 808 nm NIR irradiation produced heat to dehybrid the protector DNA/sgRNA to release sgRNA. The remaining protector DNA on the surface of gold nanorods automatically formed a hairpin structure to prevent rehybridization of DNA and released sgRNA. sgRNA then bound to Cas9 that were expressed in the pre-transfected tumor cells for gene editing after NIR irradiation.

In addition to induction of photothermal effects, light irradiation can also trigger the generation of ROS, (e.g., singlet oxygen (^1^O_2_)), in the presence of photosensitizers (e.g., chlorin e6 and verteporfin) [200, 201]. This light-triggered approach is termed photodynamic therapy (PDT) when the generated ROS concentrations are sufficiently high to kill tumor cells (cancer therapy) or bacteria (anti-bacterial therapy) [202, 203]. The generated ROS can be also harnessed to induce endo/lysosomal membrane disruption or to induce cleavage of ROS-sensitive chemical bonds for controlled payload release [204, 205]. Deng et al. [206] developed an NIR- and GSH-responsive delivery system for Cas9/sgRNA and chlorin e6 (Ce6) codelivery. Nitrilotriacetic acid-disulfanediyldipropionate-polyethyleneglycol-b-polycaprolactone (NTA-SS-PEG-PCL) copolymer self-assembled as anionic micelles to encapsulate Ce6, where polyhistidine-tagged Cas9/sgRNA bound and cationic iRGD-modified copolymer were coated on the surface. Ce6, as a photosensitizer, produced ^1^O_2_ under 671 nm irradiation to disrupt the endo/lysosomal membrane, releasing NPs to the cytosol. GSH in the cytosol broke down the disulfide bonds to liberate RNP. RNP targeting the *Nrf2* gene effectively reduced the Nrf2 protein expression in CNE-2 nasopharyngeal carcinoma cells both in vitro and in vivo in the NIR-treated group. Lyu et al. [207] also synthesized a semiconducting polymer nanotransducer (pSPN), using a thioketal moiety to link π-electron delocalized semiconducting polymer nanomaterials with PEI. The CRISPR-Cas9 plasmids were absorbed on cationic pSPN. The semiconducting polymer core under 680 nm NIR irradiation generated ROS, which cleaved thioketal bonds to release PEI and CRISPR-Cas9 plasmids. The results indicated that light-involved treatment induced 15- and 1.8-fold enhancement in gene editing in Hela cells and Hela-bearing mice, respectively.

Lanthanide-doped upconverting nanoparticles (UCNPs) are a unique type of inorganic NPs that can up-convert low-energy NIR light to high-energy ultraviolet or visible light, which allows for the initiation of photochemical reactions in deeper tissue regions that cannot be achieved by directly using UV or visible light [208, 209]. Pan et al. [210] designed an UCNPs-based system for RNP delivery (Fig. 11B). RNP was bound onto the surface of UCNPs via 4-(hydroxymethyl)-3-nitrobenzoic acid (ONA), a photosensitive molecule that can be cleaved by UV lights. Upon 980 nm NIR irradiation, UCNPs generated UV lights to degrade ONA bonds and release RNP, achieving NIR-light controlled genome editing activation both in vitro and in vivo. Based on a similar concept, Wu et al. [211] utilized UCNPs coated with photocleavable electropositive polyethylene glycol (UVP) polymers to deliver Cas9/sgPKL-1 plasmids. UVP can absorb plasmids via electrostatic interactions and undergo charge reversion upon UV lights generated by UCNPs. PKL-1 was downregulated owing to the successful genome engineering by the remote spatiotemporal control of localized Cas9/sgPKL-1 plasmid transfection.

Optogenetics is a combination of genetic and optical methods, using light to dynamically control gene-encoded proteins in cell positioning and gene expression [212]. Li et al. [213] developed a CRISPR-dCas9 optogenetic nanosystem for light-mediated gene regulation. PEI cores were attached to plasmids and were further decorated with cell-penetrating peptide- or arginine-glycine-aspartic tripeptide-linked polycaprolactone (PCL)-PEG. The construction of the plasmid was that a dCas9 gene was linked with a *calcium and integrin-binding protein 1* (cib1) gene and an *Arabidopsis* *flavoprotein cryptochrome 2* (cry2) gene fused with *histone deacetylase (HDAC) gene*. Cib1 proteins interacted with cry2 proteins in a blue light-specific manner. Once delivered into cells, sgRNA generated from the corresponding plasmids transcription was complexed with dCas9. Cry2 proteins were combined with cib1 proteins under 488 nm blue light, enabling HDAC to inhibit VEGF expression in the neovascularization area in choroid.

### Ultrasound-responsive CRISPR-Cas9 delivery

Ultrasound can also offer spatial and temporal control of genome editing. Compared to light waves, ultrasound waves can generally possess a deeper tissue penetration. Ultrasound also shows intensity- and frequency-dependent biological effects [214]. Ultrasound with high intensity can produce heat energy, while low-frequency ultrasound promotes cavitation [215]. Additionally, sonosensitizers exposed to low-intensity ultrasound and cavitation have been observed to generate ROS, which has also been termed sonodynamic therapy [216, 217].

**Fig. 12:**
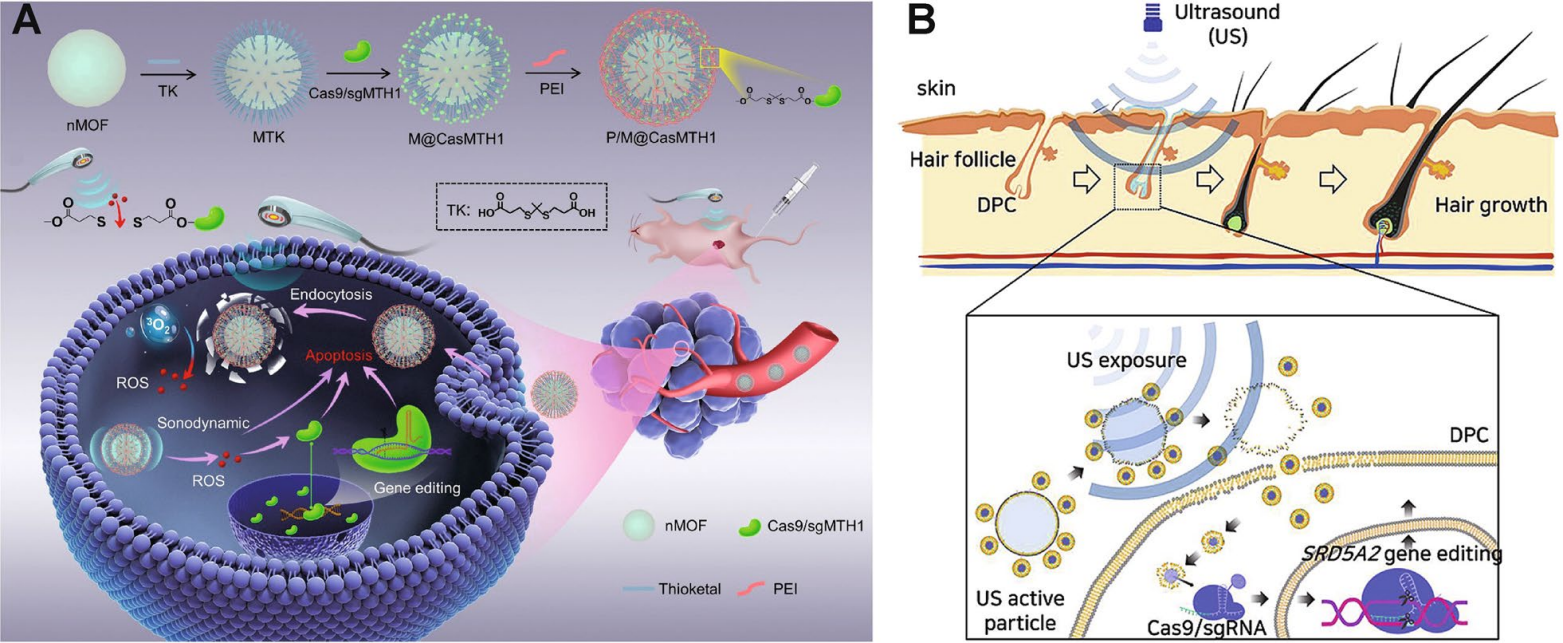
Ultrasound-responsive CRISPR-Cas9 delivery nanoformulations. **A** Schematic illustration of fabrication of the nanosonosensitizer P/M@CasMTH1 and ultrasound-triggered genome-editing-augmented SDT for tumor treatment. Reproduced with permission [218]. **B** Schematic diagram of the ultrasound flash exposure and enhancement of nanoliposome particle penetration to the dermal papilla cell cytosol through microbubble cavitation under ultrasound activation. Reproduced with permission [226]

Pu et al. [218] linked Cas9 RNPs onto the sonosensitizer-integrated nano metal-organic frameworks (nMOF) via a ROS-sensitive thioether bond (Fig. 12A). The external ultrasound triggered 5,10,15,20-tetrakis(4-carboxyphenyl)porphyrin (TCPP), a sonosensitizer in nMOF, to generate ^1^O_2_, which then cleaved the thioether bonds for effective Cas9/sgRNA release. The *MTH1* gene was knocked out in A549 human lung cancer cells, sensitizing tumor cells to ROS-induced DNA damage. In vivo results also support that this ultrasound-controlled genome editing therapy significantly inhibited tumor growth and improved surviving rate.

The ultrasound-driven nanomotors have been proved to be able to penetrate through cell membranes rapidly and have been utilized to enhance the delivery of biomacromolecules [219–222]. Hansen-Bruhn et al. [223] linked RNP onto gold nanowires (AuNWs), an ultrasound-responsive motor, *via* disulfide bonds. The obtained RNP/AuNWs were driven by ultrasound to penetrate into B16F10 cells, and RNP was released from AuNWs by intracellular GSH. RNP/AuNWs knocked out over 80% GFP in comparison with 30% in the static nanowires group without ultrasound applications.

Ultrasound can also lead to vibration of microbubbles, formation of transient pores on cell membranes, resulting in increased membrane permeability for drug delivery, which is also termed sonoporation [224, 225]. Ryu et al. [226] established an SF_6_ gas microbubble decorated with RNP-loaded nanoliposome (denoted as MB-NL(RNP)) for androgenic alopecia therapy (Fig. 12B). SF_6_ gas was bubbled in the lipid to produce microbubbles. The nanosized thiolated RNP-liposomes as the satellites were conjugated onto the microbubble core *via* disulfide bonds. The microbubbles were disrupted by the cavitation-produced sonoporation under ultrasound, thus liberating Cas9/sgRNA-liposomes into dermal papilla cells (DPCs). Steroid type II 5-alpha-reductase (SRD5A2) exists in the vertex and frontal scalp catalyzed conversion of highly-expressed testosterone (TS) to dihydrotestosterone (DHT) for DPC apoptosis, which is believed to be responsible for male-pattern baldness. RNP released from the Cas9/sgRNA-liposomes successfully knocked out the *SRD5A2* gene in DPCs, improving DPC survival. The combination of focused ultrasound with microbubbles also shows great potential for increasing the drug permeability through the blood-brain barrier (BBB) [227]. Sonoporation on BBB is non-invasive, reversible, site-specific and biocompatible [228, 229]. Yang et al. [230] prepared the lipid-polymer hybrid nanoparticle (LPHN) containing a pCas9/O6-methylguanine-DNA methyltransferase (MGMT) plasmid-absorbed PLGA core and a cationic cholesterol shell. Perfluoropropane (C_3_F_8_) was applied to solutions with lipids to produce microbubbles, and the microbubbles were linked with LPHN *via* the biotin-avidin linkage. The focused ultrasound triggered vibration of microbubbles-LPHN complexes, facilitating the opening of BBB and detachment of LPHNs from microbubbles. pCas9/MGMT downregulated the expression of MGMT to sensitize glioblastoma cells to temozolomide, enhancing the therapeutic effects of temozolomide in glioblastoma.

### Magnetic-responsive CRISPR-Cas9 delivery

**Fig. 13:**
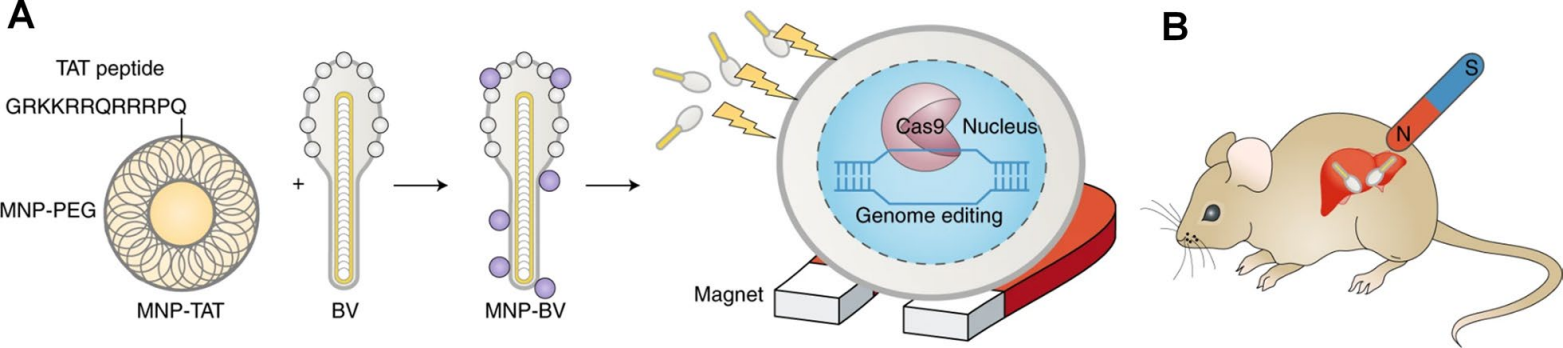
Magnetic-responsive CRISPR-Cas9 delivery nanoformulations. **A** MNPs coated with co-polymers of PEG and conjugated with a TAT peptide for facilitated interaction between MNPs and BVs. **B** Magnetic field-activating genome editing in the target organ after tail-vein injection of the MNP–BVs in mice. Reproduced with permission [232]

Magnetic-responsive drug delivery systems are typically constructed by integration of nanostructures with magnetic characteristics. With excellent tissue penetration capabilities, external magnetic field gradients allow materials to be guided towards target regions, achieving remote-controlled targeted drug delivery. In the field of genome editing, magnetism-guiding of CRISPR-Cas9 can decrease the possibility of unwanted off-target effects, making precise genome editing possible [55]. Zhu et al. [231] reported a hybrid nanoparticle-viral vector system consisting of Cas9/sgRNA DNA-containing baculoviral vectors (BVs), which was further attached to magnetic iron oxide nanoparticles (MNPs) (Fig. 13). The large size of BVs permitted a high loading capacity of DNA and provided strong yet transient gene expression due to the lack of replicate abilities in mammals. The functionalities of BVs in vivo can also be blocked by the inactivation of the complement system. The BVs inactivation could be regarded as the “off” switch to limit the systematic activities of genome editing, and the external magnetic field could be the “on” switch to facilitate the MNP-BV complex margination and entry into cells locally for tissue-specific genome editing.

Representative examples of external-stimuli responsive nanoformulations for CRISPR-Cas9 delivery are listed in Table 2. Generally, the external stimuli-responsiveness exhibits good temporal and spatial control over the function of CRISPR-Cas9 in the target sites with minimal toxicity to healthy tissues [233, 234]. One critical issue that should be carefully considered for external-stimuli nanoformations for CRISPR-Cas9 is the integration of inorganic materials. While inorganic materials exhibit unique optical, thermal or magnetic properties, there general biosafety is still under clinal investigation. Alternatively, organic or synthetic materials have also been evaluated, yet their responsiveness to the external triggers need improvements.

**Table 2:** Representative external-stimuli responsive nanoformulations for CRISPR-Cas9 delivery

Stimulus	NP composition	CRISPR-Cas9 format	Target gene locus	Applications	Administration route	Refs.
Light (808 nm)	Gold nanorods with protector DNA	sgRNA	EGFP; EMX1; PLK-1	In vitro (GFP and Cas9-expressing A549 epithelial carcinoma cells, HEK293T human embryonic kidney cells)	-	[199]
Light (980 nm)	UCNP anchored with 4-(hydroxymethyl)-3-nitrobenzoic acid and coated with PEI	RNP	EGFP; PLK-1	In vivo (A549 epithelial carcinoma in BALB/c nude mice)	Intratumoral injection	[210]
Ultrasound	Porphyrin-integrated nMOFs coated with PEI	RNP	EGFP; MTH1	In vivo (A549 epithelial carcinoma in BALB/c nude mice)	Intravenous injection	[218]
Ultrasound	Lipid nanobubble containing SF_6_ and conjugated with nanoliposome	RNP	SRD5A2	In vivo (C57/B6 mice)	Being applied to the depilated area	[226]
Magnetic	Baculoviral vectors bound with magnetic iron oxide nanoparticles	CRISPR-Cas9 plasmid	EGFP; VEGFR2	In vivo (Hepa 1–6 hepatoma in athymic nude mice and C3 knockout mice)	Intratumoral and intravenous injection	[231]

## Conclusions and outlook

Non-viral vectors for CRISPR-Cas9 have exhibited massive advantages compared with the viral vectors, including improved safety profiles, versatile loading capabilities, and decreased off-target effects. By encapsulating CRISPR-Cas9 DNA, mRNA, or RNP inside properly designed non-viral vectors, both biostability and genome-editing efficiency of CRISPR-Cas9 are enhanced. However, current non-viral nanoformulations still need improvement for precise and efficient CRISPR-Cas9 genome editing. As summarized in this Review, stimuli-responsive nanoformulations leveraging both internal biological signals and external stimuli, have been actively investigated, in hope of spatiotemporally controlling the delivery and behavior of CRISPR-Cas9 gene editors. As aforementioned, ample stimuli-responsive nanocarriers have been developed for the delivery of DNA, RNA and proteins [235, 236]. While general working mechanisms of traditional stimuli-responsive nanocarriers can be applied to CRISPR-Cas9 delivery, considering the relatively large size of CRISPR-Cas9/sgRNA components (> 160 KDa of Cas9 protein and > 30 KDa of sgRNA), the design criteria should be carefully considered [58].

While recent years have witnessed some promising results for the stimuli-responsive CRISPR-Cas9 nanoformulations, there are still several critical challenges that need thorough investigations. First, while current stimuli-responsive systems exhibit efficient and targeted CRISPR-Cas9 genome editing, the precision in selective gene editing for disease treatment is still suboptimal. Combination of both internal and external stimuli, as well as active cell/tissue targeting, could be considered. Additionally, local treatment can be a preferable method for localized diseases. Second, delivery systems integrated with donor DNA templates for gene corrections have less been investigated. This is also because co-delivery of CRISPR-Cas9 components and donor DNA templates increases the complexity of stimuli-responsive system designs, which could further raise the issues regarding biocompatibility and reproducibility. With the advance in nanotechnology and chemistry, rationalized system designs with translational and favorable manufacturing practices need to be fully considered. Third, stimuli-responsive non-viral systems can be further extended to other more recently engineered genome editing machineries, such as prime editors and base editors [7, 76, 237, 238] . Lastly, current investigations of stimuli-responsive CRISPR-Cas9 delivery systems are still limited to preclinical evaluations therefore, efforts to advance the technologies into clinical settings should be actively explored.

## Data Availability

Data sharing is not applicable to this article as no datasets were generated or analysed during the current study.
